# ATR-binding lncRNA ScaRNA2 promotes cancer resistance through facilitating efficient DNA end resection during homologous recombination repair

**DOI:** 10.1186/s13046-023-02829-4

**Published:** 2023-09-30

**Authors:** Yuanyuan Chen, Hui Shen, Tingting Liu, Kun Cao, Zhijie Wan, Zhipeng Du, Hang Wang, Yue Yu, Shengzhe Ma, Edward Lu, Wei Zhang, Jianming Cai, Fu Gao, Yanyong Yang

**Affiliations:** 1 Department of Radiation Medicine, Faculty of Naval Medicine, Naval Medical University, 800 Xiangyin Road, Shanghai, 200433 China; 2 Department of Central Laboratory, The First Affiliated Hospital of Jiaxing University, Jiaxing, China; 3 School of Public Health and Management, Wenzhou Medical University, University Town, Wenzhou, Zhejiang China; 4 Department of Colorectal Surgery, Changhai Hospital, Naval Medical University, Shanghai, China

**Keywords:** ScaRNA2, ATR, DNA end resection, Cancer resistance

## Abstract

**Background:**

Our previous study first showed that ATR-binding long noncoding RNA (lncRNA) is necessary for ATR function and promotes cancer resistance. However, the specific lncRNAs instrumental in ATR activation remain largely unclear, which limits our comprehensive understanding of this critical biological process.

**Methods:**

RNA immunoprecipitation (RIP) followed by RNA sequencing was employed to identify ATR-binding lncRNAs, which were further validated using RIP-qPCR assays. Immunofluorescence staining and Western blotting were applied to detect the activation of DNA damage repair factors. After the effect of scaRNA2 on cellular sensitivity to DNA-damaging reagents was determined, the effects of scaRNA2 on radiotherapy were investigated in patient-derived organoids and xenograft preclinical models. The clinical relevance of scaRNA2 was also validated in tissues isolated from rectal cancer patients.

**Results:**

ScaRNA2 was identified as the most enriched ATR-binding lncRNA and was found to be essential for homologous recombination (HR) mediated DNA damage repair. Furthermore, scaRNA2 knockdown abrogated the recruitment of ATR and its substrates in response to DNA damage. Mechanistically, scaRNA2 was observed to be necessary for Exo1-mediated DNA end resection and bridged the MRN complex to ATR activation. Knockdown of scaRNA2 effectively increased the sensitivity of cancer cells to multiple kinds of DNA damage-related chemoradiotherapy. Preclinically, knockdown of scaRNA2 improved the effects of radiotherapy on patient-derived organoids and xenograft models. Finally, an increase in scaRNA2 colocalized with ATR was also found in clinical patients who were resistant to radiotherapy.

**Conclusions:**

ScaRNA2 was identified as the most abundant lncRNA bound to ATR and was demonstrated to bridge DNA end resection to ATR activation; thus, it could be applied as a potent target for combined cancer treatments with chemoradiotherapy.

**Supplementary Information:**

The online version contains supplementary material available at 10.1186/s13046-023-02829-4.

## Background

Alterations of genes in DNA damage repair pathways are hallmarks of cancer. Moreover, inducing unfavourable DNA damage or blocking DNA repair is currently one of the most important strategies for cancer therapy, including radiotherapy and chemotherapy*.* Unfortunately, the abnormally stronger capacity to repair damaged DNA leads to cancer treatment resistance and treatment failure. Uncovering the underlying mechanism of enhanced DNA damage repair is of great importance for overcoming cancer treatment resistance.

Ataxia telangiectasia-mutated and Rad3-related (ATR) acts as a master kinase to phosphorylate a series of substrates in homologous recombination repair and replication stress [1]. In mammalian cells, both HR and non-homologous end joining (NHEJ) repair are often activated in response to DNA double-strand breaks (DSBs), which are the most deleterious types of DNA damage induced by radiotherapy and several chemotherapy agents [2]. In the initial stage of HR repair, DSBs are recognized, and the broken DNA ends are resected by the Mre11-Rad50-Nbs1 (MRN) complex and CtIP, together with BLM/DNA2 and Exo1. This process generates single-stranded DNA (ssDNA), which is subsequently bound and safeguarded by replication protein A2 (RPA2). RPA2-bound ssDNA recruits TOPBP1 and ATRIP to lead to ATR autophosphorylation for the activation of HR repair [3, 4]. Several ATR-targeting chemicals have been investigated in clinical trials to establish potential anticancer strategies [5, 6]. Uncovering the mechanism of ATR activation may provide novel opportunities and targets for improving cancer therapy.

Recently, RNA has emerged as a critical factor in the field of DNA damage repair [7]. Strikingly, Francia S et al. reported that RNase A treatment almost abrogated the recruitment of 53BP1, ataxia telangiectasia mutated kinase (ATM) and MDC1 to DSB sites [8]. Further investigations demonstrated that DNA damage-induced long noncoding RNA (dilncRNA) was necessary for the mobilization of protein repair factors in response to DNA damage [9]. LncRNAs such as LRIK, HITT, DSSR, and linc00312 were also demonstrated to be critical factors in the DNA damage response (DDR) by binding Ku70/80, ATM, BRCA1, and DNA-dependent protein kinase catalytic subunit (DNA-PKcs), respectively [10–13]. In particular, our recent report elucidated that lncRNA ANRIL directly binds to ATR and stabilizes the ATR protein to promote HR repair [14]. The members of ATR-binding lncRNAs during DNA repair should be identified in mammalian cells, especially in cancer cells, to potentially manage ATR-involved cancer therapy.

In our ongoing research, we performed RNA immunoprecipitation sequencing (RIP-seq) and identified Small Cajal Body-Specific RNA 2 (scaRNA2) as the most enriched lncRNA. ScaRNA2 was first shown to be related to the assembly of Cajal bodies. During this work, scaRNA2 was reported to suppress DNA-PKcs function [15]. To improve our understanding of the functions of scaRNA2 and explore potential clinical applications, we specifically studied how scaRNA2 can regulate ATR and its direct role in DNA end resection during HR repair in colorectal cancer cells, which may also provide the necessary theoretical support for targeted inhibition of HR repair in various types of cancer cells after radiotherapy in the clinic.

## Methods

### Cells and treatment

Colorectal cancer (CRC) cell lines (HCT116, HT29, WIDR and T87), lung cancer cell lines (A549, H460, H1299 and H1975), a human bronchial epithelial cell line (BEAS-2B), human glioma cell lines (U251, U373 and U87), human hepatoma cell lines (HuH7, 7721, HepG2) and human embryonic kidney 293 cells (HEK 293T) were all purchased from the Chinese Academy of Sciences Cell Bank (Shanghai, China). The culture conditions used in this study are shown in Supplementary Table S1. High glucose Dulbecco’s modified Eagle’s medium (DMEM) and RPMI-1640 medium were supplied by HyClone (Thermo Fisher, USA). All culture media were supplemented with 10% fetal bovine serum (Gibco, Thermo Fisher), 100 U/mL penicillin and 100 µg/mL phytomycin (Gibco, Thermo Fisher). All cultures were maintained at 37 °C and 5% CO_2_. For induction of DNA damage, cells were treated with camptothecin (CPT, 1 µM, Selleck) for 1 h, etoposide (ETO, 100 μg/mL, Selleck) for 4 h, or γ-irradiation. ATM inhibitor (ATMi, KU-55933), ATR inhibitor (ATRi, VE-821) and DNA-PK inhibitor (DNA-PKi, NU7441) were purchased from Selleck. During DNA damage induction by ionizing radiation (IR), CPT or ETO, the corresponding inhibitors were maintained in complete medium continuously unless indicated.

### Plasmids and sgRNA constructs

Plasmids containing full-length scaRNA2 (NR_003023) and truncated fragments were directly synthesized by OBIO Technology (Shanghai, China). The scaRNA2 overexpression sequence was cloned and inserted into the lentiviral expression vector (GL132 pSlenti-EF1-EGFP-F2A-Puro-WPRE2-CMV-MCS), and empty overexpressed lentiviral vector was used as a negative control (vector). CRISPR-mediated knockdown sequences targeting scaRNA2 were cloned and inserted into the lentiviral vector H5070 (pCLenti-U6-spgRNA v2.0-CMV-Puro-3xFlag-spCas9-WPRE). The control sgRNA lentiviral vector cells (sg-ctrl) were used as a negative control. The sequences of scaRNA2 guide RNAs are listed in Supplementary Table S3. The DRR (pLCN DSB Repair Reporter, Addgene, 98895) and DDR mCherry donor (pCAGGS DRR mCherry Donor EF1a BFP, Addgene, 98896) plasmids used in the DDR reporter assay were kind gifts from Jan Karlseder (Addgene plasmids) [16].

### RNA isolation and quantitative RT‑PCR (RT–qPCR) assay

Total RNA was extracted from cultured cells using a MagaBio plus RNA Purified Reagent Kit (Bioer Technology, Hangzhou, China) and a GenePure Pro Automatic Nucleic Acid Extraction and Purification Instrument according to the manufacturer’s instructions. The quality of the total RNA was evaluated using a NanoDrop One spectrophotometer (Thermo Fisher, USA). cDNA was synthesized by using the PrimeScript RT Master Mix Kit according to the manufacturer’s instructions (TaKaRa, Japan). qRT-PCR was conducted using a TB Green Premix Ex TaqTM PCR kit (TaKaRa, Japan) on a Quant Studio 1 (Applied Biosystems, Thermo Fisher, USA) Real-Time PCR system. Relative gene expression levels were determined using the 2^−△△CT^ method. GAPDH was used as an internal control to calculate the relative expression of lncRNAs. Each PCR amplification was performed in triplicate. All primer sequences are listed in Supplementary Table S2.

### Lentiviral transfection and stable cell line construction

The lentivirus packaging plasmids PSPAX2 and PMD2G, together with the corresponding target plasmids, were cotransfected into 293 T cells with 1 μg/mL PEI reagent. The viral supernatants were collected at 48 h and 72 h after transfection. For stably transfected cell lines with overexpression or knockdown (KD) of scaRNA2, HCT116 and HT29 cells were transduced with the harvested viral supernatants in the presence of 5 μg/mL polybrene (Yeasen Biotech, Shanghai, China) and selected with 1.5 μg/mL puromycin (Beyotime Biotech, Shanghai, China) for two weeks. The cells were then seeded at a density of 5000 cells/well into 96-well plates to obtain cell clones with stable expression. The overexpression and knockdown efficiency of scaRNA2 in these cells were examined using RT-qPCR assays.

### Irradiation

For ionizing radiation treatment, γ-irradiation was performed using a ^60^Co irradiator in the Radiation Center of Naval Medical University in China. For cellular experiments, cells were irradiated with doses ranging from 2 to 8 Gy at a dose rate of 1 Gy/min. For the animal study, tumour regions of xenograft models were subjected to local irradiation for a single dose of 15 Gy. The flow chart for the local irradiation field and shielding is shown in Figs. S14 and S15.

### The 5'-RACE and 3'-RACE techniques

5'-RACE and 3'-RACE experiments were performed using a GeneRacer™ Kit (Invitrogen, USA) with 1.0 μg of purified total RNA isolated from HCT116 cells. All RACE assays were carried out according to the manufacturer’s instructions. The primers used for 3'-RACE and 5'-RACE are listed in Supplementary Table S5.

### Fluorescence in situ hybridization (FISH) with the TSA technique

The subcellular localization of scaRNA2 was determined in HCT116 cells using an In Situ Hybridization Detection Kit (TSA technique) (Servicebio, Wuhan, China) following the manufacturer’s instructions. FISH probes against scaRNA2 were designed and synthesized using digoxigenin-labelled antisense riboprobes (Servicebio, Wuhan, China) (Table S3). Briefly, HCT116 cells were seeded on 14 × 14 Φ cover slips, fixed with 4% paraformaldehyde in DEPC at room temperature for 15 min, digested with proteinase K solution at 37 °C for 30 min, and incubated with 3% H_2_O_2_ at room temperature for 30 min. After incubation with prehybridization mix at 37 °C for 1 h, the cell slides were incubated with hybridization mix containing 1 μM scaRNA2 or U6 probes in a humidified chamber and hybridized overnight at 37 °C. The next day, the cell slides were immersed in 2 × SSC at 37 °C for 10 min, followed by two washes at 37 °C for 5 min with 1 × SSC. After 30 min of blocking with 0.1% BSA, the cell slides were labelled with digoxin working solution at 37 °C for 50 min, followed by four washes at 37 °C for 5 min with 1 × PBS. Afterwards, the cell slides were incubated with FITC-TSA working solution for 5 min in the dark. Subsequently, cell nuclei were stained with DAPI for 10 min in the dark. Fluorescence images were obtained using a Zeiss LSM primary microscope.

### Isolation of nuclear and cytoplasmic RNA fractions

The nuclear and cytosolic fractions of cellular RNA were isolated using the Cytoplasmic & Nuclear RNA Purification Kit according to the manufacturer’s instructions (Norgen Biotek, Canada). Briefly, HCT116 cells (2 × 10^6^) were lysed with 200 μL of ice-cold lysis buffer J for 5 min on ice. The lysate was transferred to a microcentrifuge tube with subsequent pelleting by spinning at 3500 × g for 10 min. The supernatant containing cytoplasmic RNA was transferred to a new tube and vortexed with 200 μL of buffer SK for 10 s. The cell pellets containing the nuclear RNA were resuspended in 400 μL of buffer SK for 10 s. Then, 200 μL of 96% ethanol was added, and the tubes were vortexed for 10 s. The sample mixtures were transferred to spin-columns to separate nuclear RNA using centrifugation (3500 g, 1 min). Next, all the columns were washed twice with 400 μL of wash solution, followed by centrifugation for 2 min to thoroughly dry the column. The samples were eluted from the columns using 50 μL of elution buffer to obtain the fractionated RNA. The concentrations of cytoplasmic and nuclear RNA were measured using a NanoDrop One spectrophotometer (Thermo Fisher, USA). Relative expression levels of U6, GAPDH and scaRNA2 were analysed using RT-qPCR.

### RNA immunoprecipitation and PCR (RIP-qPCR)

RIP was performed using a Magna RIP RNA-Binding Protein Immunoprecipitation kit (Millipore, USA) according to the manufacturer’s instructions. In brief, 2 × 10^6^ HCT116 cells were harvested and lysed with RIP lysis buffer containing protease inhibitor cocktail. The cell supernatants were incubated with magnetic beads conjugated with ATR antibody (CST, 13934s) or IgG (Millipore, AP101) for 2 h at 4 °C. RNase-free DNase I and proteinase K were consecutively used to remove DNA and protein from the RIP complex. The resulting RNA was subjected to qRT-PCR to detect the enrichment of scaRNA2.

### Colony formation assay

Cells with scaRNA2 knockdown or overexpression were seeded in 6-well plates for 24 h, followed by treatment with different doses of IR, CPT, ETO or olaparib. Cell densities and drug treatment concentrations are listed in Supplementary Table S6. After these treatments, the cells were incubated with drug-containing medium for 10–14 days. Cells were dyed with crystal violet solution (Beyotime, Shanghai, China) for 15 min after fixation with 4% paraformaldehyde for 30 min at room temperature. The images were taken with a HiCC-IB Automatic Colony Counting Analysis System (Wanshen, Hangzhou, China). Cell colonies were defined as 0.5–1 mm in diameter cell assemblies. Experiments were repeated in triplicate. Data from different experimental groups were normalized to those of their control groups.

### Flow cytometric cell apoptosis and cell cycle analysis

Cells were seeded in six-well plates at a density of 1 × 10^6^ cells/well. On the second day, cells were irradiated with 8 Gy or treated with 1 μM CPT for 1 h or 100 μg/mL ETO for 4 h. Apoptosis of cells was assessed using an Annexin V-FITC/PI Apoptosis Detection Kit (Dojindo, Japan) according to the manufacturer’s instructions. For the cell cycle assay, cells were collected and fixed with 70% ethanol overnight at 0, 4, 8, 12 and 24 h after different treatments. The cells were treated with 100 μg/mL RNase (Thermo Fisher, USA) and 40 μg/mL propidium iodide (Beyotime, Shanghai, China) for 30 min at 37 °C. The percentages of cells distributed in G0/G1-, S-, and G2/M-phase were determined using flow cytometry (Beckman, USA).

### Neutral comet assay

DNA DSBs were evaluated using a neutral single-cell gel electrophoresis assay kit (Trevigen, USA) according to the manufacturer’s instructions. In brief, negative control and scaRNA2 knockdown cells were trypsinized and resuspended at a concentration of 1 × 10^5^ cells/mL. The cell suspension was then mixed with low melting agarose (LMA) (37 °C) at a ratio of 1:10. Then, 50 μL of cell suspension was immediately pipetted onto the slide. The slides were immersed in precooled lysis buffer at 4 °C overnight and then immersed in prechilled neutralizing buffer for 30 min. After electrophoresis, the slides were immersed in DNA precipitation solution and 70% ethanol successively at room temperature for 30 min each. The slides were air dried at room temperature and stained with 100 μL of diluted SYBR® Green Gold at 4 °C for 5 min. Comet images were visualized using a fluorescence inverted microscope (LSM Primovert, ZEISS). Image quantification was performed on a minimum of five random fields per slide using CaspLab software.

### Western blot analysis

Total protein was extracted from cells using M-PER Protein Extraction Reagent (Thermo Scientific, USA) containing a proteinase inhibitor cocktail (MCE, USA), phosphatase inhibitor cocktail I (MCE, USA) and phosphatase inhibitor cocktail II (MCE, USA). The cell lysate was smashed by sonication for 15 cycles (power 6%, ultrasound for 1.5 s, interval of 1 s) in an ultrasonic cell crusher (Qiqian Electronic Technology, Shanghai, China) and centrifuged at 14,000 × g for 15 min at 4 °C. The protein concentrations were determined using a BCA protein assay kit (Beyotime, Shanghai, China). For WB analysis, 15 μg protein lysates were separated using a 10% or 12.5% SDS-PAGE gel preparation kit (Epizyme Biomedical Technology, Shanghai, China). The proteins were transferred to 0.2 μm PVDF membranes (Millipore, USA) using an eBlot™ L1 Wet Protein Film Transfer Instrument (GeneScript, China). The PVDF membranes were incubated overnight at 4 °C with the selected primary antibodies against the specific proteins (Supplementary Table S4) and then incubated with secondary antibodies for 2 h at room temperature. The western blot signals were visualized with chemiluminescence using an ECL kit (Thermo Fisher, USA) and a GelView detection system (Biolight, Guangzhou, China). Relative protein expression was quantified using ImageJ software. GAPDH was used as an internal control, and the gray value of each target protein was normalized to GAPDH. For phosphorylated proteins, grey values were normalized to those of the corresponding nonphosphorylated protein. The normalized value was calculated as the grey value of phosphorylated protein/gray value of total protein.

### Immunoprecipitation (IP)

Immunoprecipitation was performed using a Pierce™ Crosslink IP Kit (Pierce, 26147) according to the standard protocol. Briefly, negative control and scaRNA2 knockdown cells were lysed with 500 μL of IP lysis buffer with protease inhibitor cocktail plus phosphatase inhibitor cocktail (MCE) and centrifuged at 14,000 × g at 4 °C for 15 min. Total protein lysates (1 mg) were used per immunoprecipitation. Nonspecific binding of protein lysates was excluded by incubating with 80 μL of control agarose resin for 1 h and rotating at 4 °C. Antibodies against ATR, Mre11 and Exo1 or control IgG (5 μg) (Supplementary Table S4) were crosslinked with 20 μL of Protein A/G Plus Agarose for 1 h and rotated at room temperature. Then, protein lysates were loaded onto the selected antibody crosslinked columns overnight at 4 °C. On the following day, the immunoprecipitation complex was washed five times with IP lysis buffer, eluted with 40 μL of elution buffer, and analysed using western blotting along with 5% input as controls.

### RNA pulldown assay and mass spectrometry analysis

The sense and antisense strands of scaRNA2 or truncated scaRNA2 were obtained with in vitro transcription from the MB1985 pSPT18 vector (OBIO BioTech., Shanghai, China) and purified using a QIAquick Nucleotide Removal Kit (Qiagen, Germany) according to the manufacturer’s protocol. Sense and antisense RNAs were biotinylated with Biotin RNA Labeling Mix (Roche, Switzerland), T7 RNA polymerase (Roche, Switzerland) and SP6 RNA polymerase (Roche, Switzerland). The PCR products were purified using an RNeasy Mini Kit (Qiagen, Germany). The quality was verified using a NanoDrop One (Thermo Fisher, USA). Then, 3 µg of each kind of purified biotinylated transcript was heated at 90 °C for 2 min, left on ice for 2 min and incubated with 5 × Annealing Buffer (Beyotime, Shanghai, China) for 20 min at room temperature. Thereafter, the biotinylated transcripts were incubated with protein lysates from HCT116 cells (1 mg) at 4 °C for 1 h to form RNA–protein complexes. Then, the complex was incubated with Dynabeads MyOne™ (Thermo Fisher, USA) beads for 1 h at room temperature in the dark to isolate the labelled complexes from other components. Dynabeads MyOne™ beads were then washed three times with PBS and elution buffer. The proteins pulled down by sense, antisense or truncated scaRNA2 were analysed using western blotting and mass spectrometry (Thermo Fisher, USA). Mass spectra results were analysed using MaxQuant (version 1.5.5.1) software.

### NHEJ and HR reporter assay

The NHEJ and HR reporter assays were performed according to our previous study [17]. Briefly, the pLCN DSB Repair Reporter (DRR) plasmid was transfected into HeLa cells, and stable DDR expression clones were selected using 400 μg/mL neomycin. Then, the cells were transduced with scaRNA2 KD or vector lentiviruses and selected using 0.5 μg/mL puromycin. For induction of DSB formation, the I-SceI-expressing plasmid and HR donor (pCAGGS DRR mCherry Donor EF1a BFP) plasmid were cotransfected into the above selected cells. After 48 h, transfected cells were harvested and examined for the expression of GFP and mCherry by CytExpert flow cytometry (Beckman, USA).

### Chromatin fractionation

For preparation of the chromatin fractionation, a chromatin extraction kit (Abcam, UK) was utilized according to the manufacturer’s recommendations. Briefly, after different treatments, 2 × 10^6^ normal and scaRNA2 KD cells were lysed with 200 μL of lysis buffer containing protease inhibitor cocktail. The cell suspension was vortexed vigorously for 10 s and centrifuged at 5000 rpm for 5 min. The supernatant was collected and incubated with 50 µL of working extraction buffer on ice for 10 min and sonicated 2 × 20 s to increase chromatin extraction. Thereafter, the samples were centrifuged at 12,000 rpm at 4 °C for 10 min, and the supernatant was transferred to a new tube and mixed with chromatin buffer at a 1:1 ratio. The chromatin-binding proteins were analyzed using western blotting. Quantification of protein bands was analyzed using ImageJ software. Histone 3 was used as the internal control.

### Immunofluorescence (IF) staining and imaging analysis

Immunofluorescence staining assays were performed according to our previous study [17]. Briefly, negative control and scaRNA2 KD cells were seeded on circular coverslips with a diameter of 14 mm in 12-well plates at a concentration of 1 × 10^5^ cells per well. For immunostaining, cells were fixed in 4% paraformaldehyde for 15 min at different time points (30 min-24 h) after 6 Gy irradiation. Then, the coverslips were permeabilized in 0.5% Triton X-100 on ice for 10 min, followed by blockage with 5% goat serum in PBS for 1 h at room temperature. Subsequently, the coverslips were incubated at 4 °C with the appropriate primary antibodies overnight, followed by incubation with the appropriate fluorophore-conjugated secondary antibodies for 2 h in the dark at room temperature. All antibodies utilized in our study are listed in Supplementary Table S4. The nuclei were stained with 0.1 μg/mL DAPI (Beyotime, Shanghai, China) for 15 min and mounted with mounting media (Beyotime, Shanghai, China).

For the detection of RPA2, RAD51, CtIP, Mre11, DNA2, and Exo1 foci induced by IR, cell coverslips were incubated with pre-extraction buffer (25 mM HEPES pH 7.5, 50 mM NaCl, 1 mM EDTA, 3 mM MgCl_2_, 300 mM sucrose, 0.5% Triton X-100) for 5 min on ice before fixation. For the detection of ssDNA upon BrdU labelling, cells were labelled with 10 µM BrdU (Sigma, USA) for 24 h prior to exposure to IR. Before fixation, the cells were treated with fresh pre-extraction buffer as described above.

The images were photographed with a Zeiss LSM 880 confocal microscope (Zeiss, Germany) under 63 × oil objective lens magnification, and raw data were processed with Zen 2.3 software. For the quantification of foci number, approximately ten fields of view were randomly captured from distinct images.

### Patient-derived organoids and treatment

Patient-derived organoids (PDOs) were constructed to evaluate the preclinical impact of scaRNA2 knockdown on tumour growth. Tumour tissues from rectal cancer patients in the Department of Colorectal Surgery, Shanghai Changhai Hospital were used for the establishment of PDO models. Briefly, freshly resected tumour tissue was minced, rinsed with iodophor solution 3 times, cut into ~ 0.5 mm^3^ pieces and centrifuged at 200 × g for 3 min to remove the supernatant. The fragmented tissue was incubated in 4 mL of digestion buffer (Advanced DMEM F12 (Gibco, USA) containing 500 U collagenase (Gibco, USA)) on an orbital shaker at 37 °C for 2 h. After digestion, the suspension was pipetted 20 times with a 1 mL pipette tip, allowed to stand for 3 min, transferred to the supernatant and centrifuged for 1 min at 2500 rpm. Red blood cell lysis buffer (the volume of precipitation: red blood cell lysis buffer = 1:3) was added to lyse the precipitate, shaken at room temperature for 4 min, and then mixed with DPBS followed by centrifugation at 2500 rpm for 1 min. The pellet was resuspended with Matrigel (Corning, USA) on ice, and 30 µL of Matrigel resuspension was added to each well of a 48-well plate at 37 °C for 15 min. Upon complete gelation, 500 μL of Colorectal Cancer Organoid medium (Biogenous, Hangzhou, China) was added to each well, and the plates were transferred to humidified incubators. The culture media was changed every 4–5 days, and organoids were passaged every 2–4 weeks.

Cells from organoids were plated into 24-well plates after 3 days of culture and then infected with scaRNA2 knockdown or vector lentiviruses. After culture for 48 h, organoids were irradiated with 8 Gy γ-rays. Then, images were taken with a light microscope every day for up to 8 days. The average number of organoids per field was quantified.

### Tumour xenograft models and treatments

#### Cell-derived xenograft (CDX) model

In vivo subcutaneous tumour growth assays were performed in male BALB/c nude mice (4–5 weeks old) purchased from Shanghai Jihui Experimental Animal Feeding Co., Ltd. HCT116 cells were transfected with lentivirus control or sg-scaRNA2 vectors and then selected in puromycin. A total of 2.0 × 10^7^ cells were resuspended in 100 μL of culture medium and the same volume of Matrigel (Corning, USA). Each mouse was injected subcutaneously with 200 μL of cell suspension on the left and right thigh (Fig. S14A). When tumour sizes reached ~ 100 mm^3^, the mice were randomly divided into four groups: sg-ctrl group, sg-scaRNA2 group, sg-ctrl + IR group, and sg-scaRNA2 + IR group (*n* = 5). Thereafter, tumour-bearing mice received local irradiation at a dose of 15 Gy, as depicted in Fig. S15A. For the analysis of tumour growth, tumour size was measured every 3 days for a month with a digital calliper using the following formula: volume = length × width^2^ × 1/2. At the end of treatment, the mice were euthanized, and the excised tumours were fixed and serially sliced for histological and immunohistochemical analyses.

#### Patient-derived xenograft (PDX) model

For the patient-derived xenograft (PDX) model, tumour tissues from rectal cancer patients were implanted into nude mice and established by LIDE Biotech Co., Shanghai, China. Tumour tissues from mice bearing the passage 3 PDX (COPF161282) were injected subcutaneously into the dorsal flank of male BALB/c nude mice. When the tumours reached 100–200 mm^3^, 100 μL (10^8^ IU/mL) of sg-ctrl or sg-scaRNA2 lentivirus was intratumorally injected at multiple points. The lentiviruses were injected every two days three times (Fig. S14B), and then, the mice were randomly divided into four groups as in the PDX models (*n* = 4). Tumours in the flank of PDXs were locally irradiated with a ^60^Co irradiator at a dose of 15 Gy, and the abdomen of mice was covered with custom lead jigs (Fig. S15B). The tumour size was monitored every 3 days for a month. Tumour volume was calculated in accordance with the following formula: volume = length × width^2^ × 1/2. At the endpoint, the mice were euthanized, and the tumours were excised, measured, weighed and fixed for analysis with histology and immunohistochemistry.

### Histology and immunohistochemistry

Tumour tissues from CDX and PDX were collected and fixed with 4% paraformaldehyde overnight and sectioned into slides at a thickness of 5 μm. For histological analysis, slides were stained with haematoxylin and eosin (H&E). For immunohistochemistry, slides were deparaffinized, rehydrated, and treated with 3% H_2_O_2_ for 25 min in the dark. After extensive washing, the slides were blocked with 3% BSA in PBS for 30 min and incubated overnight at 4 °C with the indicated primary antibodies, followed by incubation with the appropriate fluorophore-conjugated secondary antibodies. Antibody information is shown in Supplemental Table S4.

TUNEL staining was conducted to evaluate apoptosis in resected tissue according to the manufacturer’s instructions (Servicebio, Wuhan, China). Briefly, after deparaffinization and antigen retrieval, sections were incubated with 1% Triton X-100 in PBS for 20 min at room temperature. Then, the sections were incubated with reaction solution containing 1 µL of TdT, 5 µL of dUTP and 50 µL of equilibration buffer at 37 °C for 2 h in a humidified box. After washing with PBS, the sections were stained with DAPI for 10 min at room temperature. The slides were scanned using a panoramic slice scanner (3DHISTECH, Hungary). ImageJ software was used for quantitation of TUNEL-positive cells.

### Clinical patient sample collection and tissue microarray

Between May 2016 and October 2019, 80 patients with locally advanced rectal cancer were enrolled from the Department of Colorectal Surgery, Shanghai Changhai Hospital. The inclusion criteria were a single primary lesion, completion of standard neoadjuvant chemoradiotherapy, surgical resection, and survival beyond 1 month post-surgery. Resected tumour and adjacent tissues were harvested, paraffin-embedded, and assembled into TMAs. Neoadjuvant chemoradiotherapy was administered at a total dose of 50 Gy, divided into 2 Gy per fraction. Typically, surgery was scheduled 6–8 weeks following the completion of preoperative radiotherapy. Tumour regression post-chemoradiotherapy was assessed using the tumour regression grade (TRG) as follows: TRG 0 (complete regression): absence of tumour cells under microscopy; TRG 1 (close to complete regression): scattered or minimal tumour cells observed microscopically. TRG 2 (partial regression): significant regression with more residual tumours than single or small tumour cells; TRG 3 (poor/no regression): extensive residual tumour without significant regression.

### Immunohistochemical staining of TMAs

ScaRNA2 and ATR expression in the TMAs was detected with FISH and immunohistochemistry. The procedures for the scaRNA2 probe hybridization were consistent with the above FISH method. After FITC-TSA incubation, the slides were incubated at 4 °C overnight with ATR primary antibody (Abcam, ab289363, 1:50) in a humidified chamber, followed by the corresponding goat anti-rabbit Cy3 secondary antibody for 1 h at 37 °C in the dark. Image information on the tissue slice was scanned using a panoramic slice scanner (3DHISTECH, Hungary). CaseViewer 2.4 software was employed at 1–400 × arbitrary magnification for observation.

### Clinical analysis for tissue microarray

The TMA plug-in in the Halo v3.0.311.314 analytical software (Indica Labs, USA) was used to set the chip tissue point diameter and the number of rows and columns, and the software automatically generated the number (Table S7). The Indica Labs-Highplex FLv3.1.0 module was used to quantify the number of positive cells and total cells in the target area of each slice. Positive cell rate (%) = number of positive cells/total number of cells. Positive cell density (cells/mm^2^) = the number of positive cells (cells/analysis area in mm^2^). We used the minimum *P* value method to obtain the cut-off value based on the log-rank test. Based on this established threshold, we classified patients into two distinct groups according to their scaRNA2 levels, denoted as “high” or “low”. The survival outcomes of patients from these two groups were analyzed with Kaplan–Meier survival curves based on scaRNA2 expression levels (Table S7).

### Statistical analysis

All statistical analyses were performed using GraphPad Prism 8.0 software. Quantitative data are presented as the means ± standard deviations (SD). For experiments including only two groups, means were evaluated using a two-tailed unpaired Student’s *t* test. For multiple group comparisons, data were analysed using one-way or two-way ANOVA followed by Tukey’s multiple-comparisons test or Dunnett’s test. *P* < 0.05 was considered statistically significant. All experiments were performed at least three times independently.

## Results

### ScaRNA2 is identified as the top enriched ATR-binding lncRNA

**Fig. 1:**
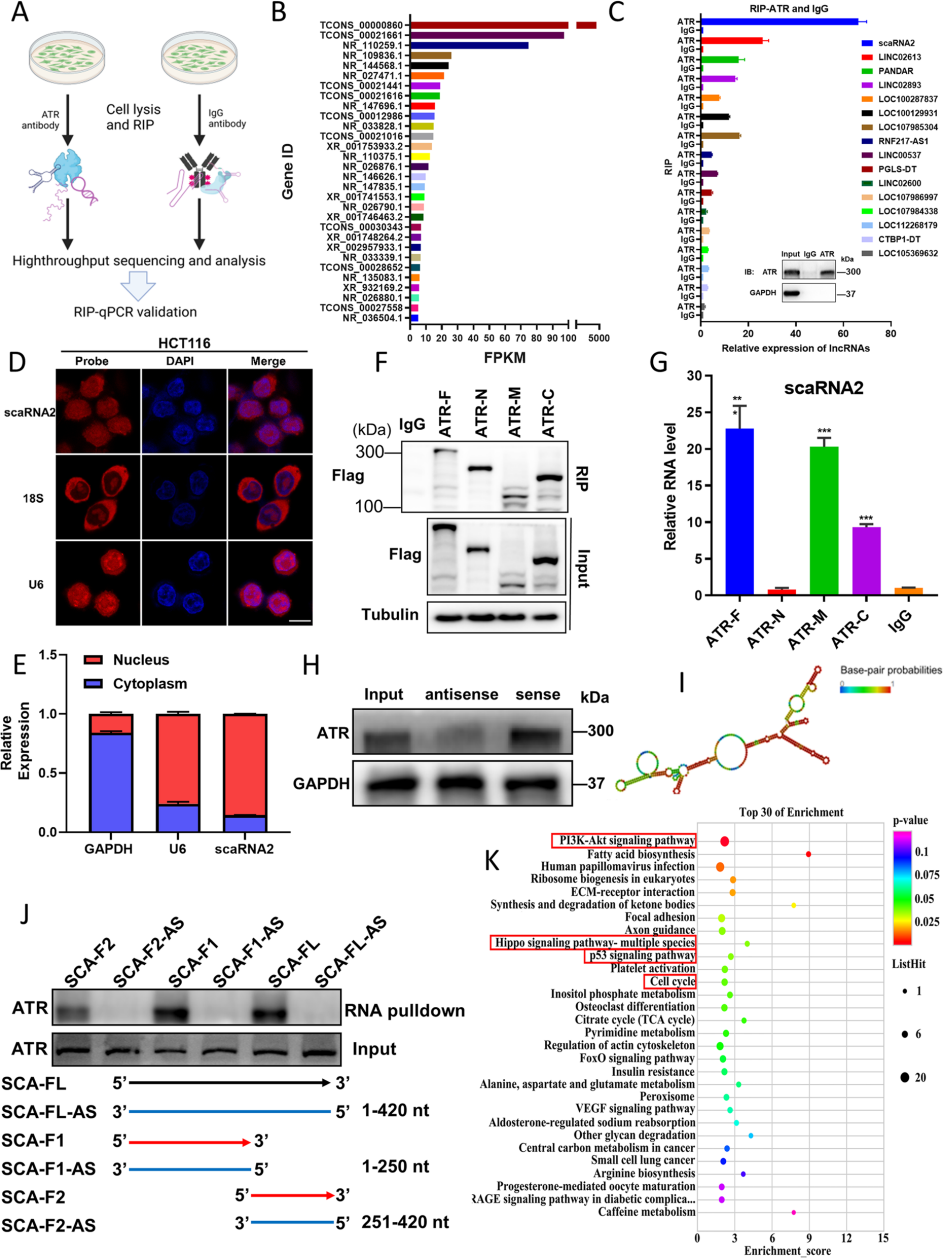
Characterization of scaRNA2 as an ATR-binding lncRNA and its influence on the transcriptome. **A** Schematic view of RIP and sequencing analysis in HCT116 cell lysate with an ATR-specific antibody. **B** The top 30 ATR-binding lncRNAs are shown in the column graph based on the expression level from RIP-seq. **C** ATR-binding lncRNAs were confirmed with a RIP-qPCR assay in HCT116 cells with an ATR-specific antibody. A representative image of immunoblotted ATR from RIP experiments is shown. **D** Representative images of RNA FISH staining with a specific probe for scaRNA2 in HCT116 cells. U6 was used as a positive marker for nuclear lncRNAs. Scale bar: 20 μm. **E** Relative scaRNA2 expression was determined with nuclear and cytoplasmic RNA extracted from HCT116 cells. **F** Representative images of ATR fragments immunoblotted with Flag antibody in RIP experiments. ATR-F: full-length; ATR-N: ATR N-terminal; ATR-M: ATR middle region; ATR-C: ATR C-terminal. **G** The relative expression of scaRNA2 measured with an RT-PCR assay in the ATR RIP complex. ***P < 0.001 compared with the IgG group. **H** Representative images of immunoblotted ATR from the RNA pulldown complex using biotin-labelled sense and antisense scaRNA2. **I** The structure of scaRNA2 predicted using RNAfold software. **J** Representative images of ATR from RNA pulldown experiments with scaRNA2 full-length (SCA-FL), scaRNA2 fragment 1 (SCA-F1), scaRNA2 fragment 2 (SCA-F2), and the respective antisense sequences. **K** GO analysis of the key signalling pathways affected by scaRNA2

To screen for potential ATR-binding lncRNAs, we performed RIP and RNA sequencing assays with the lysate of HCT116 cells by using an ATR-specific antibody (Fig. 1A). Among the detected candidates, the top 30 enriched lncRNAs (shown in Fig. 1B) were further validated with RIP-qPCR analysis compared to the IgG control (Fig. 1C, Table S2). Notably, lncRNA TCONS_00000860 was identified as the most prominently enriched lncRNA bound to ATR, prompting an in-depth investigation of its physical characteristics and potential function. The sequence of this ATR-binding lncRNA was aligned to scaRNA2 through a RACE experiment (Fig. S1A). The RNA FISH assay showed that scaRNA2 was mainly located in the nucleus (Fig. 1D, Fig. S1G), which was further confirmed in the RT–qPCR assay by using a cytoplasm/nucleus separation method (Fig. 1E).

To delineate the specific domain of ATR for scaRNA2 binding, we constructed Flag-tagged truncated ATRs, including the N-terminal (ATR-N), middle (ATR-M), and C-terminal (ATR-C) regions, as previously described [15]. Then, RIP-qPCR experiments revealed that scaRNA2 binds to ATR at the M region and C-terminal region (Fig. 1F, G). The direct interaction between scaRNA2 and ATR was further confirmed with an RNA pulldown experiment (Fig. 1H). Using RNA fold software, we predicted the secondary structure of scaRNA2 (Fig. 1I) and observed more scaRNA2 binding with the ATR protein at its 3' region (Fig. 1J).

Then, scaRNA2 expression across different cancer cell lines was detected by RT-PCR, which revealed that scaRNA2 was highly expressed in HCT116 and HT29 colorectal cancer cells (Fig. S1B). Next, RNA sequencing was performed to detect the global influence of scaRNA2 on the transcriptome in HCT116 cells. A subset of the differentially expressed genes is shown in the HEAT map (Fig. 1K). Gene Ontology (GO) and KEGG analyses of differentially expressed genes (DEGs) revealed that PI3K-Akt, p53 signalling, cell cycle, and HIPPO signalling pathways were predominantly affected in the scaRNA2 KD cells (Fig. 1K, Fig. S2A, S2B). Collectively, our data underscore scaRNA2 as the predominant ATR-binding lncRNA, suggesting its pivotal role in ATR activation and DNA damage repair.

### ScaRNA2 is necessary for HR-mediated DNA damage repair

**Fig. 2:**
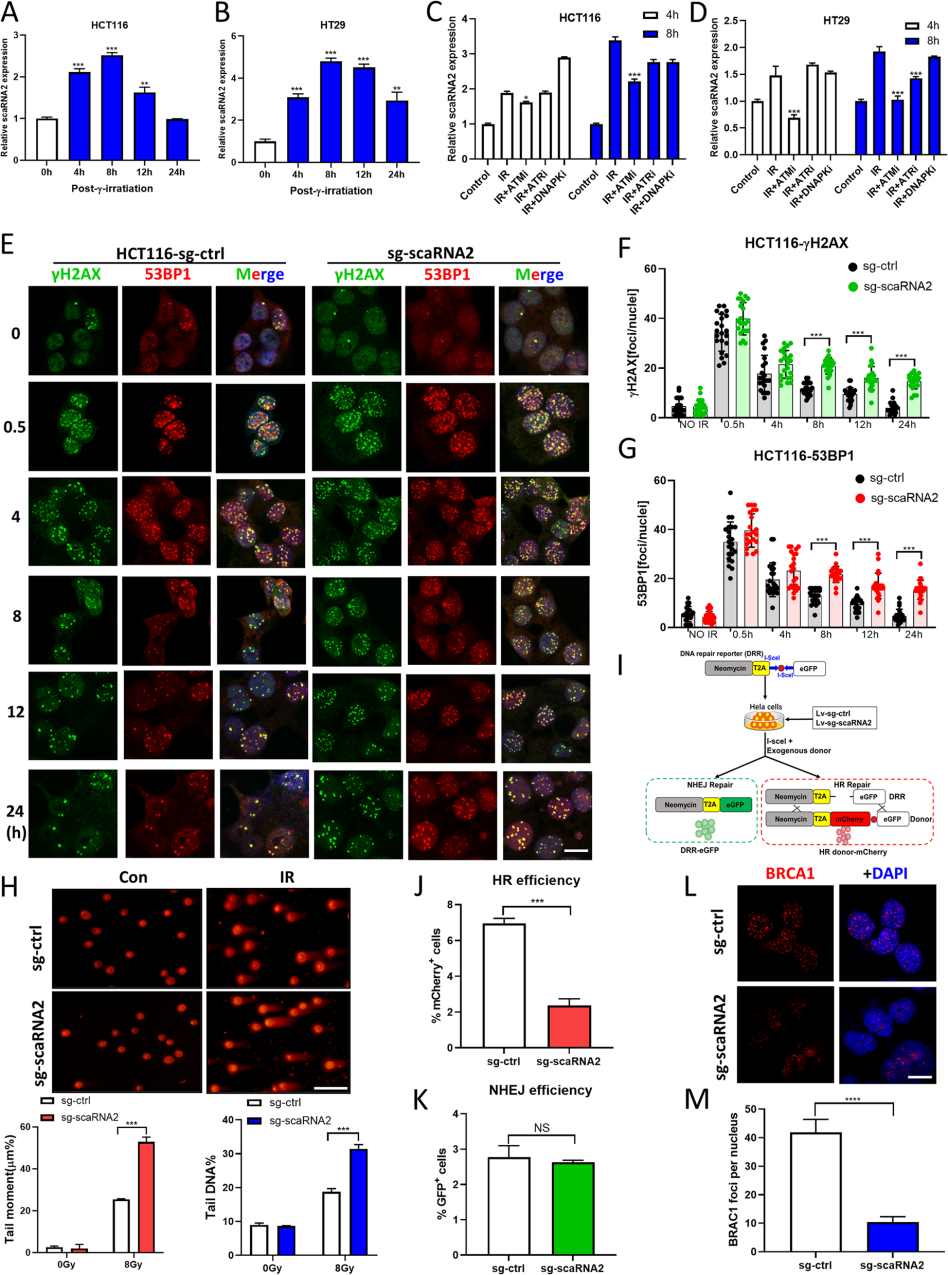
ScaRNA2 is necessary for HR-mediated DNA damage repair. **A**-**B** Relative expression of scaRNA2 in HCT116 and HT29 cells detected with an RT–PCR assay at 0, 4, 8, 12 and 24 h after irradiation. **C**-**D** Column graphs of scaRNA2 expression in HCT116 and HT29 cells treated with an ATM inhibitor (KU55933, 10 μM), ATR inhibitor (VE821, 10 μM) and DNA-PKcs inhibitor (NU7441, 10 μM) for 2 h before irradiation. **E** Immunofluorescence staining of γH2AX and 53BP1 in irradiated sg-ctrl and scaRNA2 knockdown HCT116 cells at 0, 0.5, 4, 8, 12 and 24 h after irradiation (6 Gy). Scale bar: 20 μm. **F**-**G** Quantification of γH2AX and 53BP1 foci at each time point after irradiation. ***P < 0.001, *P < 0.05 compared with the sg-ctrl group. Ns, nonsignificant. **H** Representative images and quantification of comet tails (tail moment and tail DNA) at 8 h in irradiated sg-ctrl and scaRNA2 knockdown cells (8 Gy). Scale bar: 50 μm. **I** Schematic view of NHEJ and HR reporter assays. **J**-**K** NHEJ repair and HR repair efficacies were detected with the reporter system and quantified with flow cytometry. **L**-**M** Representative images and quantification of BRCA1 foci measured at 8 h in irradiated sg-ctrl and scaRNA2 knockdown HCT116 cells (6 Gy)

In both HCT116 and HT29 cells, the expression level of scaRNA2 was dramatically induced by DNA damaging reagents, including IR (Fig. 2A, B), CPT (Fig. S3A, S3B) and ETO (Fig. S3C, S3D). Using ATM (Ku55933), ATR (VE821) or a DNA-PK inhibitor (NU7441), we found that the upregulation of scaRNA2 in HCT116 and HT29 cells was mainly dependent on ATM/ATR (Fig. 2C, D; Fig. S2E-H). Then, the role of scaRNA2 in DNA damage repair was investigated with γH2AX foci and comet assays. As expected, more γH2AX foci and 53BP1 foci were detected at later time points (8–24 h) in both the scaRNA2 KD HCT116 (Fig. 2E, F, G) and scaRNA2 KD HT29 cells (Fig. S4A, S4B, S4C). In the neutral DNA comet assay, significantly more tailed DNA and tail moment-representing unrepaired DNA damage was detected in the scaRNA2 KD HCT116 (Fig. 2H) and scaRNA2 KD HT29 cells (Fig. S5A, S5B, S5C). These data showed that scaRNA2 is necessary for efficient DNA damage repair.

To delineate the specific role of scaRNA2 in different repair mechanisms, we compared the efficiencies of NHEJ and HR repairs using a pLCN DSB Repair Reporter assay following scaRNA2 knockdown [16]. Remarkably, the efficacy of HR repair was dramatically reduced in the scaRNA2 KD cells, while NHEJ efficacy was not affected (Fig. 2I, J, K). This defect in HR repair was further confirmed by reduced BRCA1 foci in the scaRNA2 KD cells (Fig. 2L, M). Thus, the multi-step evidences in our study indicate that scaRNA2 is indispensable for HR-mediated DNA damage repair.

### ScaRNA2 is required for Mre11- and Exo1-mediated DNA end resection

**Fig. 3:**
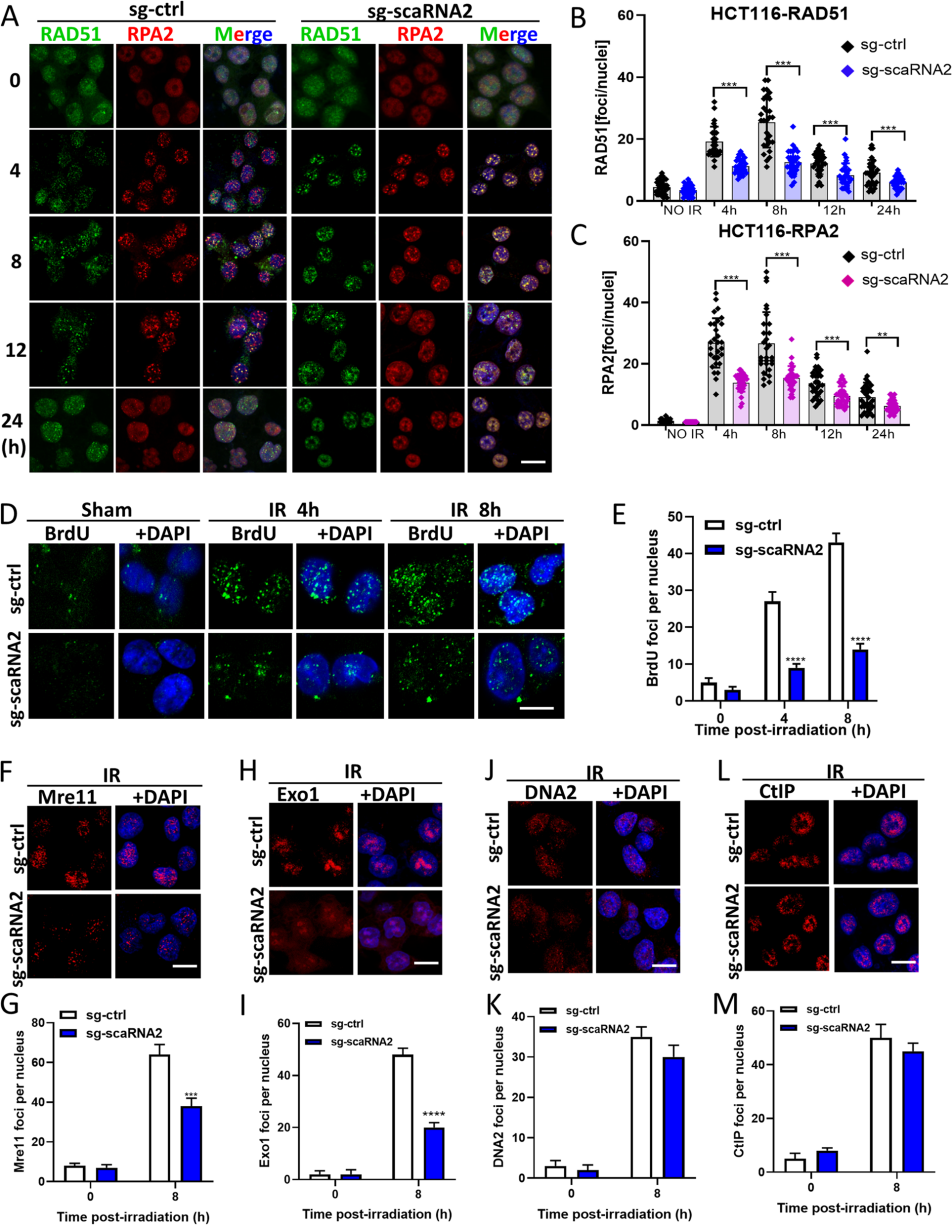
ScaRNA2 is required for efficient Mre11- and Exo1-mediated DNA end resection. **A** Immunofluorescence staining of RAD51 and RPA2 foci in irradiated sg-ctrl and scaRNA2 knockdown HCT116 cells at the indicated time points after irradiation (6 Gy). Scale bar: 20 μm. **B**, **C** The numbers of RAD51 foci and RPA2 foci per nucleus in irradiated HCT116 cells with/without scaRNA2 knockdown. ***P < 0.001, **P < 0.01 compared with the sg-ctrl group. **D**, **E** After labelling with BrdU for 24 h, sg-ctrl and scaRNA2 knockdown HCT116 cells were irradiated at a dose of 6 Gy. At 4 and 8 h later, the cells were fixed and stained for BrdU together with DAPI. Scale bar: 20 μm. The number of BrdU foci per nucleus was counted for statistical analysis. ****P < 0.0001 compared with the sg-ctrl group at the same radiation dose. **F**-**M** The main endonucleases for DNA end resection accounting for DSB level, including Mre11 (**F**, **G**), Exo1 (**H**, **I**), DNA2 (**J**, **K**) and CtIP (**L**, **M**), were detected with immunofluorescence staining in HCT116 cells at 8 h after 6 Gy irradiation. Quantification of the foci number in each group is provided as a column graph. ****P < 0.0001, ***P < 0.001 compared with the sg-ctrl group

To explore the mechanism of HR defects in scaRNA2 KD cells, we carried out immunostaining experiments with antibodies against RAD51 and RPA2. Compared to that of negative control cells, the average number of RAD51 foci was greatly reduced in both the scaRNA2 KD HCT116 (Fig. 3A, B) and scaRNA2 KD HT29 cells (Fig. S6A, S6B). The number of RPA2 foci was also decreased in the scaRNA2 KD HCT116 (Fig. 3A, C) and scaRNA2 KD HT29 cells (Fig. S6A, S6C). The impairment of RPA2 recruitment indicates a potential deficit in ssDNA generated during DNA end resection. To verify whether DNA end resection occurs normally in scaRNA2 KD cells, we used a BrdU assay to specifically label ssDNA as previously reported [18]. The number of BrdU foci was significantly decreased in the scaRNA2 KD HCT116 cells (Fig. 3D, E), suggesting a possible role of scaRNA2 in DNA end resection. Based on this, we carried out immunostaining experiments to analyse the recruitment of other MRN complex members as well as key endonucleases, including Exo1, BLM and DNA2. Intriguingly, the recruitment of Mre11 and Exo1 was dramatically impaired in the scaRNA2 KD cells (Fig. 3F-I). Moreover, the lack of scaRNA2 did not affect DNA2 and CtIP recruitment after irradiation (Fig. 3J-M). Collectively, these findings implied that scaRNA2 is necessary for the recruitment of Mre11 and Exo1 to the DSBs in DNA end resection.

### Defects in DNA end resection impair scaRNA2-mediated ATR activation

**Fig. 4:**
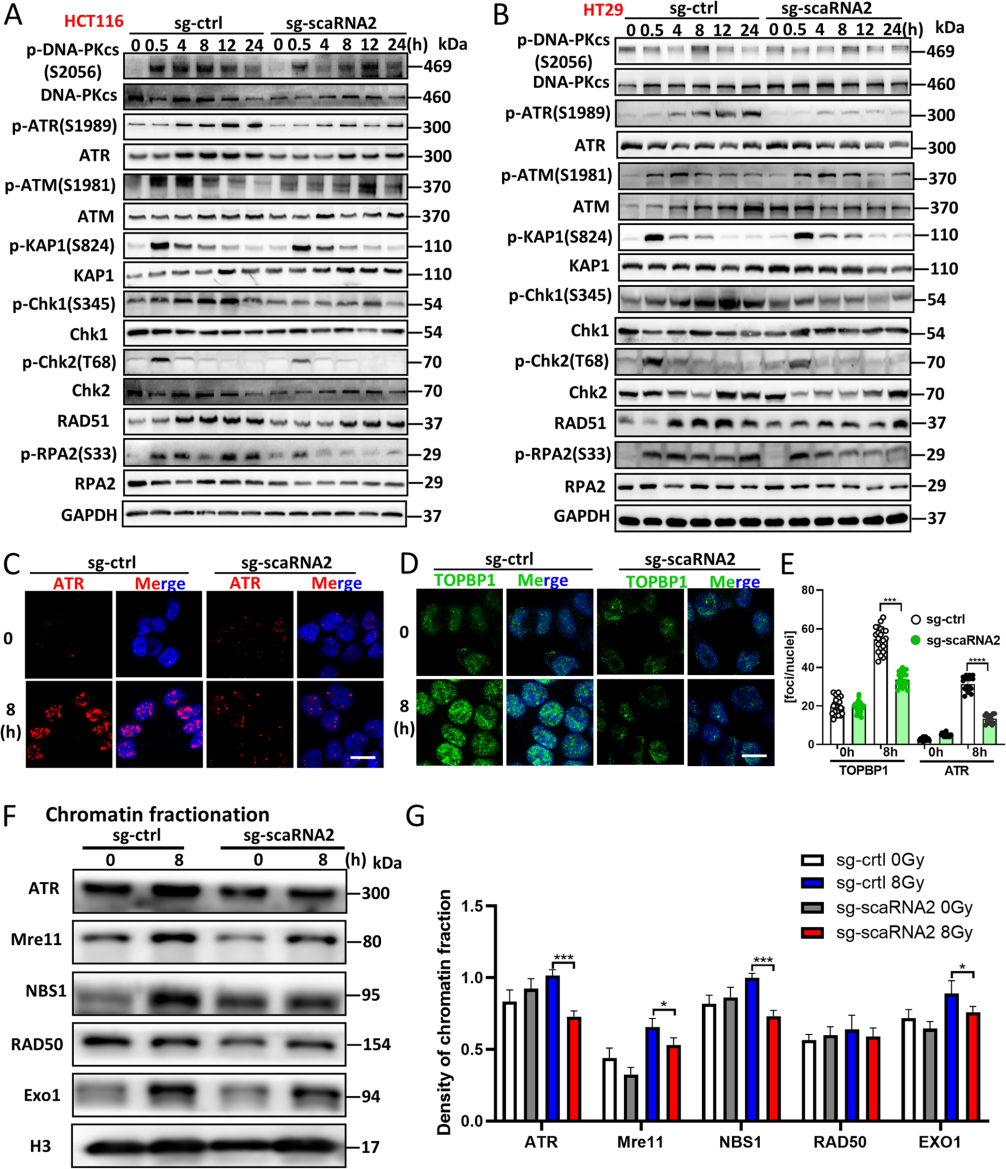
ScaRNA2 knockdown resulted in ATR inactivation, accounting for HR defects. **A**-**B** HCT116 cells and HT29 cells with/without scaRNA2 knockdown were treated with 8 Gy irradiation, and proteins involved in the DNA damage response were detected with western blotting assays at the indicated time points. The specific sites for phosphorylated proteins are indicated. GAPDH was used as a negative control. **C**, **D** Activation of ATR and TOPBP1 in HCT116 cells was detected with immunofluorescence staining at 8 h after 8 Gy irradiation. Scale bar: 20 μm. **E** The numbers of ATR foci and TOPBP1 foci per nucleus in irradiated HCT116 cells with/without scaRNA2 knockdown. ****P < 0.0001, ***P < 0.001 compared with the sg-ctrl group. **F** sg-ctrl and scaRNA2 knockdown HCT116 cells were treated with 8 Gy irradiation, and the chromatin fractionation protein was extracted. Proteins including ATR, the MRN complex and Exo1 were detected using a western blot analysis. **G** The raw density of each protein in the indicated four groups was quantified and statistically analysed. ***P < 0.001, *P < 0.05 compared with the sg-ctrl group

The MRN complex and DNA end resection were reported to be critical for ATR activation [19, 20]. Next, the DNA damage response was determined to investigate whether scaRNA2-related DNA end resection is involved in specific signalling pathways. After scaRNA2 knockdown in both HCT116 and HT29 cells, the phosphorylation of ATR at S1989 and phosphorylation of Chk1 at S345 were inactivated after irradiation (Fig. 4A, B; Fig. S7C, S7D). Moreover, consistent ATR signalling defects were observed in the scaRNA2 KD HCT116 cells after DNA damage was induced by ETO and CPT treatment (Fig. S7A, S7B, S7F, S7I). Furthermore, the phosphorylation of ATM and its substrates, including p-KAP1 and p-Chk2, remained unchanged in normal cells (Fig. 4A, B; Fig. S7A, S7B). In response to DNA damage, ATR is dynamically mobilized and recruited to DSB sites via the coordinated action of TOPBP1 and ATRIP [1]. However, the average number of ATR foci in the scaRNA2 KD cells was significantly reduced compared to that of the negative control cells (Fig. 4C, E). As a direct activator of ATR, TOPBP1 was significantly inhibited for recruitment in the scaRNA2 KD cells (Fig. 4D, E). The recruitment of resection factors to damaged DNA was further confirmed with chromatin fractionation assays. In the scaRNA2 KD cells, the binding of Mre11, Exo1 and ATR-related factors to damaged DNA was dramatically reduced after DNA damage (Fig. 4F, G). Collectively, our observations demonstrate that the depletion of scaRNA2 hampers Exo1-mediated DNA end resection, which subsequently inhibits the activation of ATR.

### ScaRNA2 bridges the DNA end resection complex to ATR activation

**Fig. 5:**
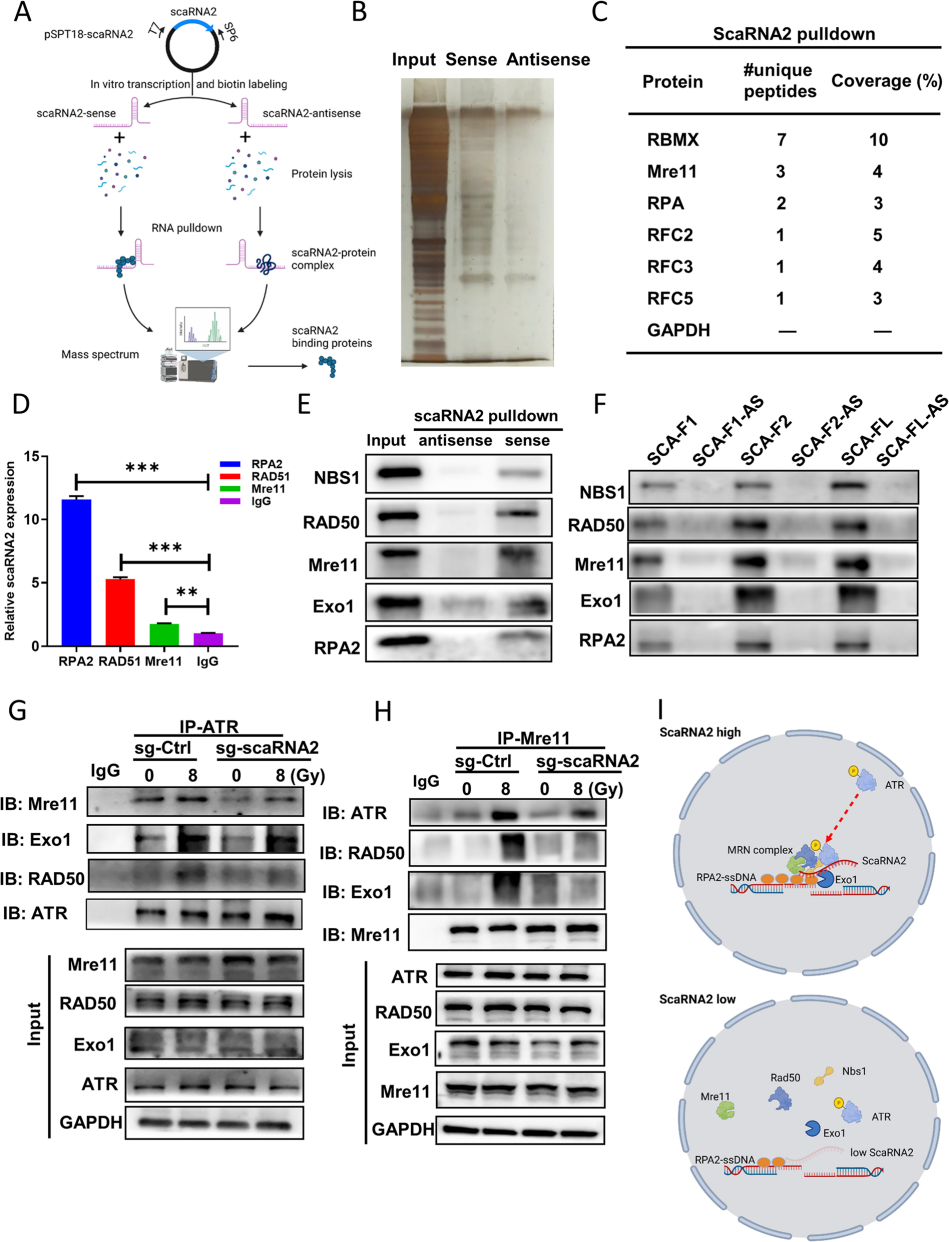
ScaRNA2 bridges RPA-ssDNA to the assembly of the MRN complex and ATR recruitment. **A** Schematic diagram of RNA pulldown and mass spectrometry to identify scaRNA2 binding proteins. **B**-**C** Representative images of silver staining of proteins after electrophoretic separation. Proteins including RBMX, Mre11, RPA, and replication factors such as RFC2-5 were identified. The number of peptides and coverage are indicated. **D** The binding of scaRNA2 with RPA2, Mre11 and RAD51 was determined with RIP experiments with a specific antibody in irradiated HCT116 cells. ***P < 0.001, **P < 0.01 compared with the IgG group. **E** An RNA pulldown assay was performed with biotin-labelled scaRNA2 and protein lysis from irradiated HCT116 cells. The MRN complex, RPA2 and Exo1 were detected using a western blot analysis. **F** The binding of scaRNA2 fragments with the MRN complex was determined with biotin-labelled scaRNA2 FL, scaRNA2 F1 and scaRNA2 F2 transcripts. **G**-**H** In sg-ctrl and scaRNA2 knockdown HCT116 cells, immunoprecipitation was conducted with ATR (**G**) or Mre11 (**H**) antibody to detect the binding between MRN members and ATR at 8 h after 8 Gy irradiation. **I** Schematic model of MRN assembly and ATR recruitment after irradiation in the presence and absence of scaRNA2

To explore the precise role of scaRNA2 in DNA resection, we performed mass spectrometry to screen scaRNA2-binding proteins following the RNA pulldown experiment (Fig. 5A). First, scaRNA2-bound proteins were subjected to silver staining after SDS-PAGE (Fig. 5B). After analysing the mass data, we identified several ATR-related proteins involved in DNA damage repair and replication: RBMX, Mre11, RPA2, and RFC2/3/5 (Fig. 5C). Then, interactions between scaRNA2 and key factors in HR repair were confirmed with RNA immunoprecipitation with RPA2-, Mre11-, or RAD51-specific antibodies. Of these, RPA2-bound lncRNAs displayed the most enrichment with scaRNA2 (Fig. 5D). RNA pulldown experiments using sense scaRNA2 transcripts confirmed its interactions with Mre11 and Exo1, with the strongest binding observed at its 3' end (Fig. 5E, F).

Co-IP was performed to ascertain whether the physical binding of ATR and resection factors is influenced by scaRNA2. The Co-IP data with the ATR antibody showed that the binding of ATR and Mre11 was reduced in the scaRNA2 KD cells (Fig. 5G). In the IP complex with the Mre11 antibody, less ATR and Exo1 protein was detected (Fig. 5H). The binding mode of scaRNA2 in conjunction with the DNA end resection complex and ATR is illustrated in Fig. 5I. These data suggested that scaRNA2 potentially functions as a scaffold bridging the DNA end resection complex to ATR upon DNA damage.

### Knockdown of scaRNA2 increases sensitivity to radiotherapy in cancer cells and patient-derived organoid (PDO) model

**Fig. 6:**
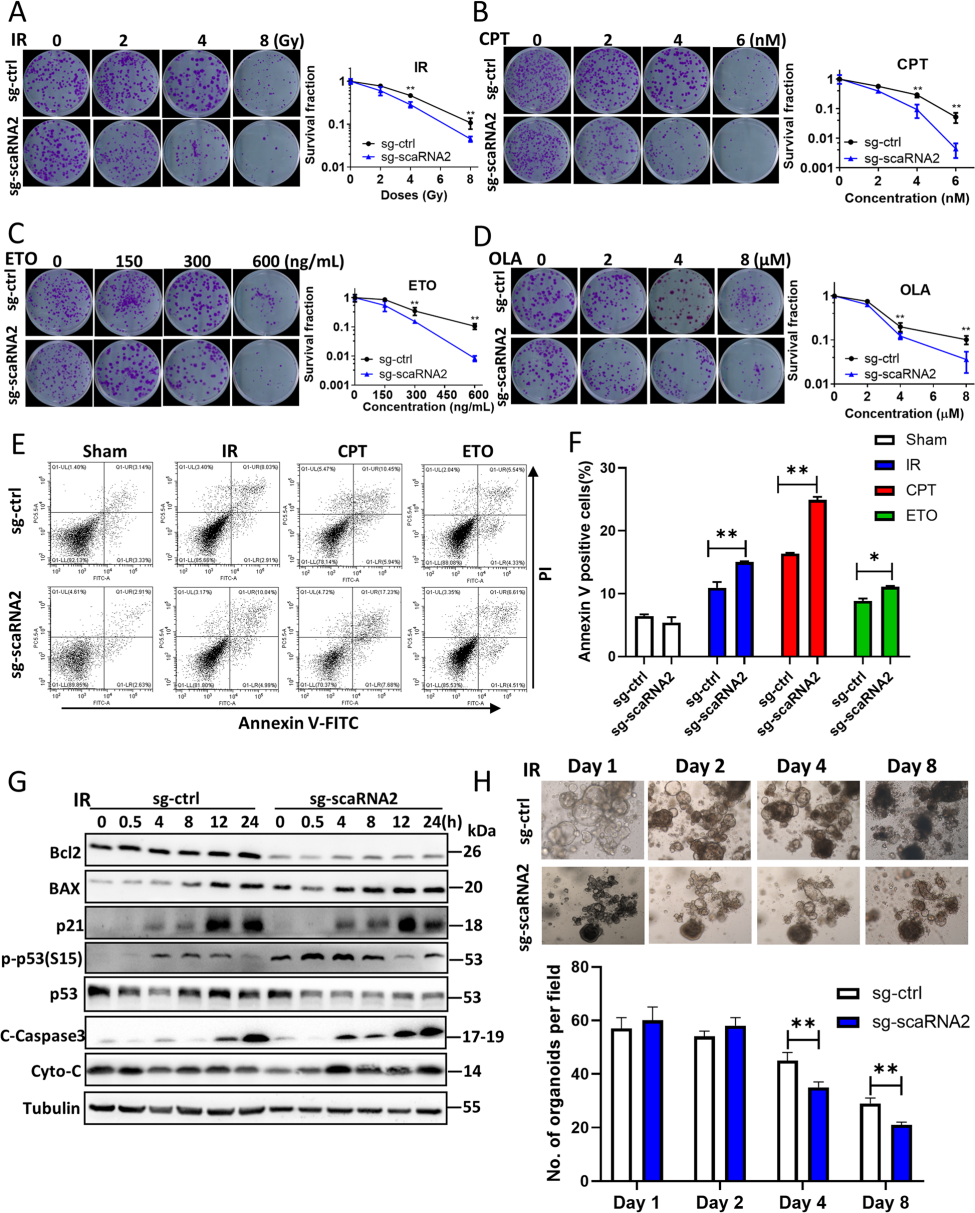
Knockdown of scaRNA2 increased sensitivity to chemoradiotherapy in cancer cells. **A**-**D** Survival of sg-ctrl and scaRNA2 knockdown HCT116 cells after irradiation (**A**), CPT (**B**), etoposide (**C**) or olaparib (**D**) treatment at the indicated doses (concentrations) was determined using a colony formation assay. **P < 0.01 compared with the sg-ctrl group at the same dose of treatment. **E**, **F** Cell apoptosis in sg-ctrl and scaRNA2 knockdown HCT116 cells after irradiation, CPT and ETO treatment was measured with flow cytometry using an Annexin V/PI double staining method. Quantification of Annexin V-positive cells is shown in the column graph. **P < 0.01, *P < 0.05 between the indicated two groups. **G** Proteins involved in the cell apoptosis signalling pathway were determined by western blotting in sg-ctrl and scaRNA2 HCT116 knockdown cells at the indicated times after 8 Gy irradiation. **H** Rectal cancer patient-derived organoids (PDOs) were cultured and infected with sg-ctrl and sg-scaRNA2 lentiviruses. Images were taken on Days 1–8 after irradiation at a magnification of 40X. The number of PDOs was calculated in each field from different groups. **P < 0.01 between the indicated two groups

Disrupting DNA end resection and HR repair by depleting scaRNA2 provides a novel opportunity for improving neoadjuvant chemoradiotherapy of cancer. In the colony formation assay, the cell survival fraction was significantly reduced in the scaRNA2 KD HCT116 cells after exposure to IR, CPT, ETO or olaparib treatment (Fig. 6A-D). Conversely, overexpression of scaRNA2 significantly increased cellular resistance to these DNA damage treatments (Fig. S8A-S8H). Furthermore, significantly elevated cell apoptosis was detected in the scaRNA2 knockdown cells after IR, CPT and ETO treatment compared with the control parental cells (Fig. 6E, F). In the scaRNA2 KD cells, earlier and increased activation of proapoptotic Bax, p21 and C-Caspase3 was observed (Fig. 6G). Proapoptotic proteins were also inhibited in the scaRNA2-overexpressing HCT116 cells in the IR groups as well as chemotherapeutic drugs (Fig. S9A-C). In addition, results of the cell cycle assay indicated that the numbers of scaRNA2 KD cells that progressed to G2/M arrest after multiple types of DNA damage decreased significantly (Figs. S10A-F and S11A-F). Phosphorylation of ATR-Chk1 downstream factors, including CDC25A (S124), CDC25C (S216), and CDC2 (T15), was attenuated in the scaRNA2 KD cells (Figs. S12A-D; S13A-D).

To investigate the preclinical relevance of scaRNA2, we established PDO models with surgically resected colorectal tumours. Knockdown of scaRNA2 in CRC PDOs significantly inhibited the growth of PDOs when combined with irradiation, suggesting a critical role of scaRNA2 in overcoming resistance to radiotherapy (Fig. 6H).

### Knockdown of scaRNA2 improves the efficacy of radiotherapy in CRC xenografts

**Fig. 7:**
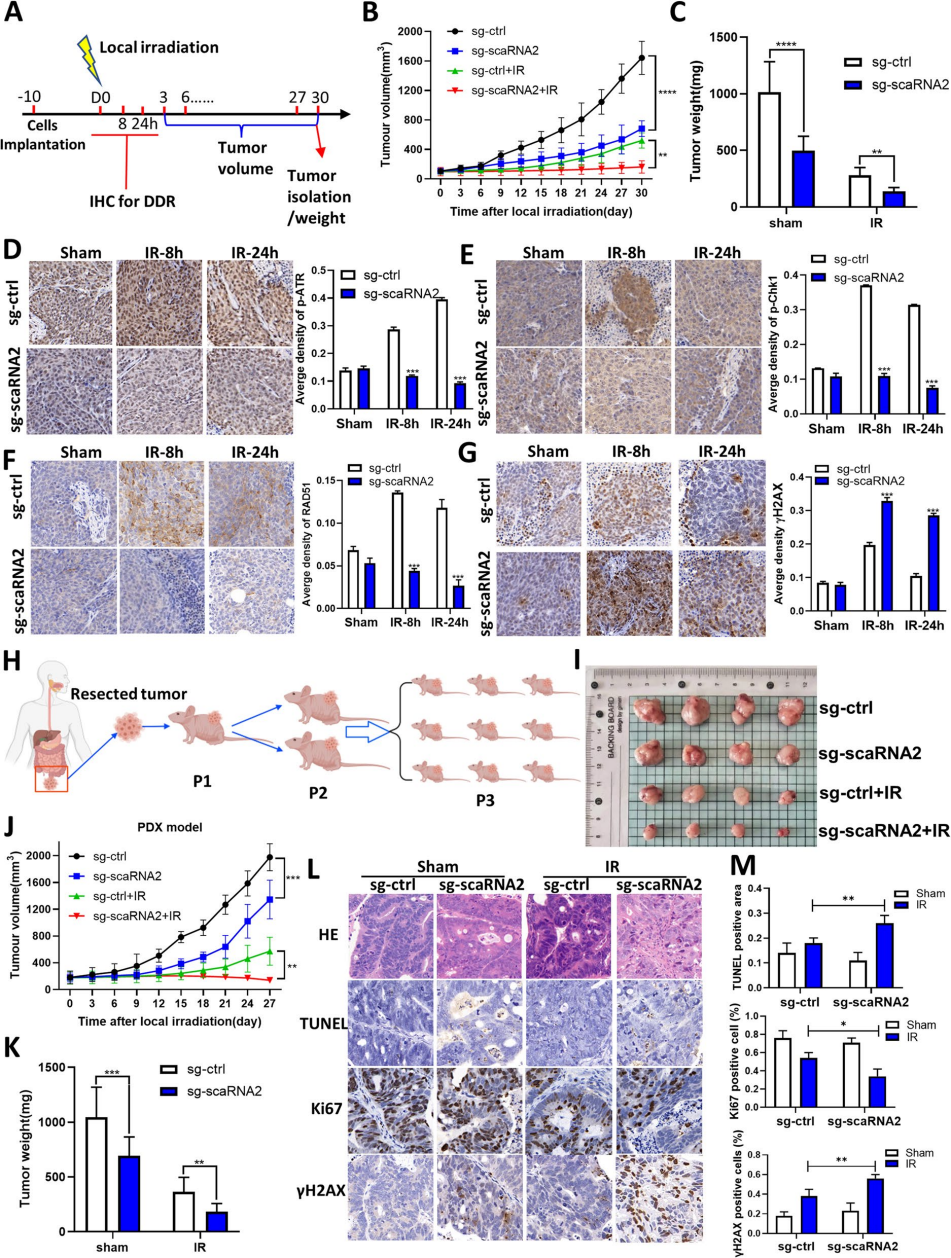
Knockdown of scaRNA2 sensitized colorectal cancer to radiotherapy. Cell-derived xenografts (CDXs, A-G) and patient-derived xenografts (PDXs, H-M) were used to determine the influence of scaRNA2 knockdown on sensitivity to radiotherapy. **A** Schedule of the implantation of sg-ctrl and scaRNA2 knockdown HCT116 cells, local irradiation and measurement time points. **B**-**C** Tumour volumes in irradiated or unirradiated tumours were recorded every two days after local irradiation at a dose of 15 Gy. Tumour weight in the indicated four groups was measured on Day 30 after irradiation. ***P < 0.001 between the sg-ctrl + IR and sg-scaRNA2 + IR groups. **D**-**G** Immunohistochemical staining of p-ATR, p-Chk1, RAD51 and γH2AX in tumour tissues isolated from sg-ctrl and scaRNA2 knockdown tumours at 0, 8 and 24 h after irradiation. Quantification of the average density per field was performed for each group. ***P < 0.001 between the sg-ctrl + IR and sg-scaRNA2 + IR groups at each time point. **H** Schematic diagram depicting the establishment of the PDX model from rectal cancer patients. **I** Representative image of tumours in the sg-ctrl, sg-ctrl + IR, sg-scaRNA2 and sg-scaRNA2 + IR groups isolated on the 30^th^ day after irradiation. **J**, **K** Tumour volumes in irradiated or unirradiated tumours were recorded every three days after local irradiation at a dose of 15 Gy. Tumour weight in the indicated four groups was measured on Day 30 after irradiation. ***P < 0.001 between the sg-ctrl + IR and sg-scaRNA2 + IR groups. **L**, **M** HE and immunohistochemical staining of TUNEL, Ki67 and γH2AX in tumour tissues isolated from negative control and scaRNA2 knockdown tumours after irradiation. Quantification of the average density per field was also performed in each group. ***P < 0.001 between the sg-ctrl + IR and sg-scaRNA2 + IR groups at each time point

We further established both CDX model and PDX models to investigate the therapeutic potential of the combined treatments of scaRNA2 knockdown and radiotherapy in vivo. In the CDX model, when the tumours grew to 100 mm^3^, they were exposed to local irradiation for 15 Gy at a dose rate of 1 Gy/min (Fig. 7A; Figs. S14, S15). The growth curve revealed that tumour progression was significantly inhibited in the scaRNA2 KD group compared to the single radiotherapy group (Figs. 7B, S16B). At the endpoint of 30 days after irradiation, the tumour weight in the scaRNA2 KD group was significantly reduced (Fig. 7C). IHC staining and quantitative analysis revealed that the phosphorylation of ATR and Chk1 and the expression of Rad51 were inhibited in tumours derived from the scaRNA2 KD cells (Fig. 7D-F). More unrepaired DNA damage (γH2AX) was also observed at both 8 h and 24 h after local irradiation (Fig. 7G).

To explore the potential clinical application of inhibiting scaRNA2 combined with radiotherapy, we established PDX models using tumour tissues isolated from clinical patients (Fig. 7H). The PDX-bearing mice were intratumorally injected with lentivirus-packaged scaRNA2 KD plasmid and exposed to local irradiation 48 h later. The knockdown efficacy was validated (Fig. S16A). We found that scaRNA2 KD significantly inhibited tumour growth after local irradiation (Fig. 7I, J). The tumour weights in the scaRNA2 KD group were also reduced compared with those in the negative control group (Fig. 7K). TUNEL and Ki67 staining experiments showed that more cell apoptosis and fewer proliferating cells were observed in the scaRNA2 KD PDX models (Fig. 7L, M). More DNA damage, as shown by γH2AX staining, was observed in the PDXs injected with scaRNA2 KD lentivirus (Fig. 7L, M). These findings underscore the potential of scaRNA2 as a novel target for combinational therapeutic strategies, particularly in conjunction with radiotherapy.

### High levels of ScaRNA2 predict poor outcomes for patients with radioresistant tumours

**Fig. 8:**
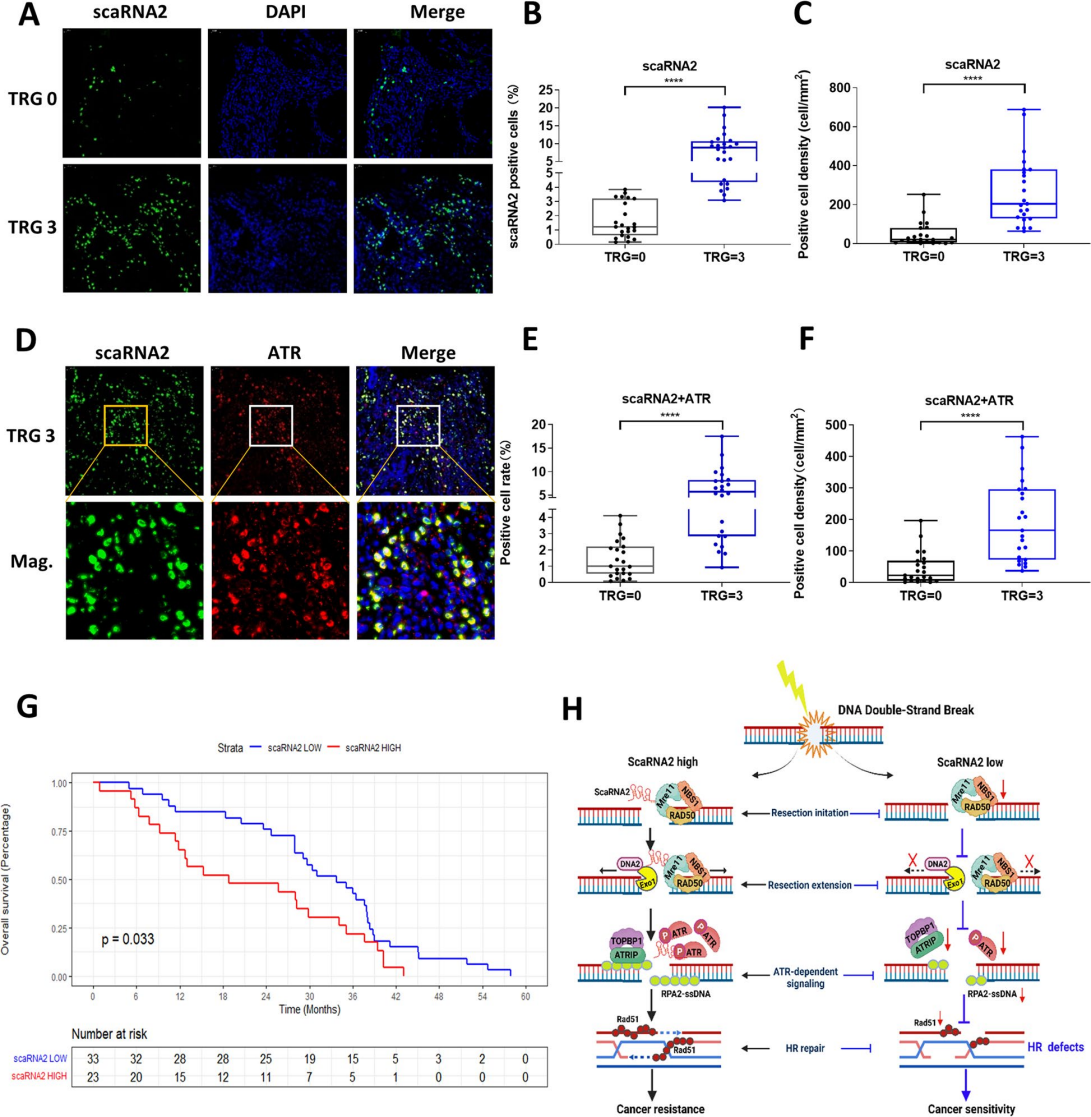
Elevation of scaRNA2 predicts poor response to radiotherapy in rectal cancer. **A** Tumour tissues from 80 rectal cancer patients were surgically resected, and radiosensitivity was classified based on the TRG score. TRG0, radiosensitive group, TRG3, radioresistant group. Scale bar: 5 μm. **B**-**C** Quantification of scaRNA2-positive cells and positive cell density in each section of tumours from the radiosensitive group and radioresistant group. ****P < 0.0001 between the two groups. **D** Immunofluorescence staining of ATR (red) and scaRNA2 FISH (green) were performed on the same slide to visualize the colocalization of ATR and scaRNA2. Scale bar: 5 μm. **E**–**F** The colocalized ATR- and scaRNA2-positive cells and the positive cell density were quantified in the radiosensitive group and radioresistant group. ****P < 0.0001 between the two groups. **G** Kaplan–Meier survival analysis of clinical patients from the microarray of rectal cancer patients. ***P < 0.001. **H** A proposed possible mechanistic model of how scaRNA2 regulates DNA damage repair and cancer resistance by facilitating DNA end resection

Neoadjuvant radiotherapy is a standard treatment for locally advanced rectal cancer [21]. After confirming the potential role of scaRNA2 as a novel target in the adjuvant therapy of rectal cancer, we sought to determine whether it correlates with radiation resistance in clinical patients. Eighty patients with locally advanced rectal cancer participated in this study. According to their tumour regression grading (TRG) scores, all patients were classified into a radiosensitive group (TRG0-2) and a radioresistant group (TRG3). A tissue array was prepared and subjected to RNA FISH staining of scaRNA2 (Fig. S17). Quantitative analysis of RNA FISH revealed that scaRNA2 was significantly upregulated in radioresistant cancer tissues compared with that in radiosensitive tissues (Fig. 8A, B, C). Co-IHC staining of ATR together with the scaRNA2 FISH probe revealed colocalization of scaRNA2 and ATR in rectal cancer tissues (Fig. 8D). The double-positive cells of ATR and scaRNA2 were found to be more prevalent in radioresistant tumours (Fig. 8E, F). Based on Kaplan–Meier survival analysis, we found that patients with scaRNA2^high^ had significantly shorter disease-free survival (DFS, *P* = 0.033, Fig. 8G). Collectively, our findings indicate that scaRNA2 could serve as a potent biomarker and present a critical therapeutic target for circumventing resistance to radiotherapy or chemotherapy in cancer. (Fig. 8H).

## Discussion

In this study, we identified scaRNA2 as the most enriched lncRNA bound to ATR and demonstrated its key function in ATR activation and HR repair. Mechanistic investigation showed that scaRNA2 bridges Exo1-mediated DSB resection to ATR activation and DNA damage repair. Knockdown of scaRNA2 combined with radiotherapy is a potent strategy for cancer therapy in vitro and in preclinical models.

DNA damage repair is crucial for maintaining genome stability and is also the core mechanism of cancer resistance [3]. Shortly after DNA damage occurs, key repair factors, including DNA-PKcs, ATM, ATR, and their substrates, are recruited to DSB sites following H2AX phosphorylation [22]. Recently, the essential roles of lncRNAs in DNA damage repair have drawn increasing attention. The mechanisms of lncRNAs in regulating DNA damage repair include the ceRNA mechanism, the cis mechanism, and the modulation of protein functions via direct interaction [23]. In our previous study, the first ATR-binding lncRNA was found to maintain ATR protein stability, suggesting that binding with lncRNAs is critical for ATR function [17]. Continuing with the previous findings, we identified the most enriched lncRNA, which was identified as scaRNA2 after RACE experiments. Further analysis revealed that scaRNA2 binds with ATR at the N-terminus and M-region with its 3' region. This finding is consistent with our previous work, demonstrating that the N-terminus of ATR contains a lncRNA binding domain. Furthermore, this finding aligns with Hu’s observations, which highlight that the HEAT domain of ATM also binds with lncRNA, and a characteristic HEAT domain was similarly observed in ATR [13]. The RNA pulldown experiment with complementary scaRNA2 fragments also showed increased scaRNA2 binding with the ATR protein at its 3' region. We hypothesize that this could be attributed to the secondary structure of scaRNA2, which may dictate specific binding interactions. The specific binding modalities continue to be an area of ongoing investigation.

ScaRNA is a group of lncRNAs assembling Cajal bodies and has been reported to be relevant to cancer resistance [14, 24]. In our study, we also found that scaRNA2 was responsive to DNA damage treatments, which implies a possible role in DNA damage repair. Then, through kinase inhibitors targeting ATM/ATR/DNA-PKcs, we demonstrated that scaRNA2 upregulation was mainly dependent on ATM/ATR. Nevertheless, high concentrations of inhibitors targeting these three key kinases might lead to off-target effects. Therefore, we will pursue further investigations to validate our findings in other models. Through γH2AX foci and comet assays, we found that scaRNA2 knockdown significantly inhibits the repair of DNA damage resulting from various kinds of stimuli. The DDR reporter assay further showed that scaRNA2 primarily modulates HR repair rather than NHEJ. Additionally, upon scaRNA2 knockdown, we noted suppression of the phosphorylation and mobilization of ATR. During the preparation of this work, Marianne et al. reported that scaRNA2 contributes to HR-mediated DNA damage repair by inhibiting DNA-PKcs [25]. Our findings provide another possible mechanism of scaRNA2 in HR repair via its direct binding with ATR.

In response to DNA damage, ATR is often activated by TOPBP1 and ATRIP in the presence of RPA-bound ssDNA generated through DNA end resection [26, 27]. DNA end resection is also a critical switch for HR repair, differing from NHEJ repair [28]. Through BrdU foci, it was observed that loss of scaRNA2 led to a reduced level of DSB resection. We screened possible resection factors, including Mre11, Exo1, DNA2, BLM and CtIP, by IF staining assay using confocal microscopy [29]. Our data show that Exo1 foci are reduced in scaRNA2 KD cells, suggesting that Exo1 may be a critical endonuclease required for DNA end resection. Moreover, ATR activation was found to be abrogated in Exo1 knockout cells, suggesting that scaRNA2-mediated activation of ATR is related to Exo1. These data identify a possible role for scaRNA2 in bridging Exo1 to ATR activation by affecting DSB resection [30]. However, the exact mechanism of scaRNA2 in Exo1 recruitment and ATR activation remains to be investigated.

Moreover, our findings using several preclinical models provide a clinical translational opportunity for cancer therapy. Knockdown of scaRNA2 extensively increased cellular sensitivity to IR and other chemotherapy drugs, including CPT, ETO and olaparib. ScaRNA2 knockdown combined with radiotherapy strongly inhibited the growth of PDOs, CDXs and PDXs. Currently, targeting ATR is also considered a potent strategy to overcome cancer resistance, and several ATR inhibitors are in clinical trials [5, 6]. Targeting ATR also alters the microenvironment and enhances the activation of the c-GAS-Sting pathway [31, 32]. Our study provides a potent therapeutic target of the ATR-scaRNA2 complex for overcoming cancer resistance. Further experiments with 80 rectal cancer patients showed that scaRNA2 expression is significantly correlated with radiosensitivity.

Although our study provides a possible opportunity for combined cancer treatment with radiotherapy, there are still some shortcomings that need to be resolved. First, the detailed mechanism by which scaRNA2 bridges DNA end resection to ATR-mediated HR requires deeper exploration. Second, the overall DNA damage-induced lncRNAs bound to ATR need to be identified. Finally, novel strategies targeting the ATR-scaRNA2 complex need to be developed for use in clinical cancer treatment.

In conclusion, we identified and characterized scaRNA2 as the most enriched lncRNA bound to the ATR protein. Knockdown of scaRNA2 inhibited DNA damage repair by abrogating ATR activation and HR repair mediated by DSB resection. Knockdown of scaRNA2 increased cellular sensitivity to chemoradiotherapy and increases therapeutic efficacy in both PDO and PDX models. Beyond its therapeutic targeting potential, the increased scaRNA2 levels in the radioresistant tumours suggest a potential biomarker for cancer resistance.

### Supplementary Information

**Additional file 1: Fig. S1.** Characterization of scaRNA2 and construction of stably overexpression and knockdown cell lines. **Fig. S2.** Bioinformatics analysis of RNA sequencing in normal and scaRNA2 knockdown cells. **Fig. S3.** ScaRNA2 is responsive to DNA damage in colorectal cancer cells. **Fig. S4.** ScaRNA2 knockdown impairs DNA damage repair kinetics in HT29 cells. **Fig. S5.** ScaRNA2 knockdown resulted in more unrepair DNA damages in HT29 cells. **Fig. S6.** ScaRNA2 knockdown inhibited the recruitment of RPA2 and RAD51. **Fig. S7.** ScaRNA2 is necessary for the DNA damage responses after CPT and ETO treatment. **Fig. S8.** Overexpression of scaRNA2 significantly increased cellular resistance to DNA damage treatments. **Fig. S9.** Overexpression of scaRNA2 inhibits apoptosis activation by DNA damage. **Fig. S10.** ScaRNA2 knockdown inhibited DNA damage checkpoint activation in HCT116 cells. **Fig. S11.** ScaRNA2 knockdown inhibited DNA damage checkpoint activation in HT29 cells. **Fig. S12.** Knockdown of scaRNA2 inhibits the progression of cell cycle into G2/M after irradiation. **Fig. S13.** Overexpression of scaRNA2 promoted cell cycle progression after irradiation. **Fig. S14.** Schematic illustration of local irradiation and lentivirus transfection in cell-derived xenografts (CDX, A) and patient-derived xenografts (PDX, B). **Fig. S15.** Schematic diagram of local irradiation field and shielding of cell-derived xenografts (CDX, A) and patient-derived xenografts (PDX, B). **Fig. S16.** Knockdown of scaRNA2 sensitized colorectal cancer to radiotherapy. **Fig. S17.** Scanned image of tissues microarray including CRC patients included in our study. RNA FISH and immunofluorescence staining were performed to detect the expression of scaRNA2 and ATR, respectively. **Table S1.** The cells and culture conditions. **Table S2.** List of PCR primers used in the study. **Table S3.** List of Sequences of sgRNAs and primers employed in this study. **Table S4.** List of antibodies used in the study. **Table S5.** Sequences of and primers employed in RACE experiment. **Table S6.** Cell densities and drug concentrations in Colony Formation Assay. **Table S7.** Quantification of scaRNA2 positive cells in rectal cancer tissues/adjacent tissues.

## Data Availability

The datasets used and analysed during the current study are available within the manuscript and its additional files.

## Abbreviations

ScaRNA2: Small Cajal body-specific RNA 2 (scaRNA2)
ssDNA: Single stranded DNA
DSBs: DNA double strand breaks
DDR: DNA damage response
NHEJ: Nonhomologous end joining
HR: Homologous recombination
ATR: Ataxia telangiectasia-mutated and Rad3-related
ATM: Ataxia telangiectasia mutated
DNA-PKcs: DNA-dependent protein kinase catalytic subunit
MRN: Mre11-Rad50-Nbs1
RPA2: Replication protein A2
PDOs: Patient-derived organoids
CDX: Cell-derived xenograft
PDX: Patient-derived xenograft
TMAs: Tissue microarrays
