# Supplementary material for: ATR-binding lncRNA ScaRNA2 promotes cancer resistance through facilitating efficient DNA end resection during homologous recombination repair
Source: J Exp Clin Cancer Res. 2023 Sep 30;42:256. doi: 10.1186/s13046-023-02829-4 (PMC10542231; doi:10.1186/s13046-023-02829-4)
Supplement: Supplementary file 1 — Additional file 1: Fig. S1. Characterization of scaRNA2 and construction of stably overexpression and knockdown cell lines. Fig. S2. Bioinformatics analysis of RNA sequencing in normal and scaRNA2 knockdown cells. Fig. S3. ScaRNA2 is responsive to DNA damage in colorectal cancer cells. Fig. S4. ScaRNA2 knockdown impairs DNA damage repair kinetics in HT29 cells. Fig. S5. ScaRNA2 knockdown resulted in more unrepair DNA damages in HT29 cells. Fig. S6. ScaRNA2 knockdown inhibited the recruitment of RPA2 and RAD51. Fig. S7. ScaRNA2 is necessary for the DNA damage responses after CPT and ETO treatment. Fig. S8. Overexpression of scaRNA2 significantly increased cellular resistance to DNA damage treatments. Fig. S9. Overexpression of scaRNA2 inhibits apoptosis activation by DNA damage. Fig. S10. ScaRNA2 knockdown inhibited DNA damage checkpoint activation in HCT116 cells. Fig. S11. ScaRNA2 knockdown inhibited DNA damage checkpoint activation in HT29 cells. Fig. S12. Knockdown of scaRNA2 inhibits the progression of cell cycle into G2/M after irradiation. Fig. S13. Overexpression of scaRNA2 promoted cell cycle progression after irradiation. Fig. S14. Schematic illustration of local irradiation and lentivirus transfection in cell-derived xenografts (CDX, A) and patient-derived xenografts (PDX, B). Fig. S15. Schematic diagram of local irradiation field and shielding of cell-derived xenografts (CDX, A) and patient-derived xenografts (PDX, B). Fig. S16. Knockdown of scaRNA2 sensitized colorectal cancer to radiotherapy. Fig. S17. Scanned image of tissues microarray including CRC patients included in our study. RNA FISH and immunofluorescence staining were performed to detect the expression of scaRNA2 and ATR, respectively. Table S1. The cells and culture conditions. Table S2. List of PCR primers used in the study. Table S3. List of Sequences of sgRNAs and primers employed in this study. Table S4. List of antibodies used in the study. Table S5. Sequences of and primers employed in [file 13046_2023_2829_MOESM1_ESM.pdf]

Supplementary Information

ATR-binding lncRNA ScaRNA2 promotes cancer resistance through facilitating efficient DNA end resection during homologous recombination repair

Yuanyuan Chen[#1], Hui Shen [#1,4], Tingting Liu[#1], Kun Cao [1], Zhijie Wan[1], Zhipeng Du[3], Hang Wang[1], Yue Yu[2], Shengzhe Ma[2], Edward Lu[1], Wei Zhang*[2], JIanming Cai*[3], Fu Gao*[1], Yanyong Yang*[1]

[#]Authors contributed equally to this work.

1 Department of Radiation Medicine, Faculty of Naval Medicine, Naval Medical University, 800, Xiangyin Road, 200433, Shanghai, China;

2 Department of Colorectal Surgery, Changhai Hospital, Naval Medical University, Shanghai, China;

3 School of Public Health and Management, Wenzhou Medical University, University Town, Wenzhou, Zhejiang, China;

4 Department of Central Laboratory, The First Affiliated Hospital of Jiaxing University, Jiaxing, China.

Running title: ScaRNA2 promotes HR repair through regulating DSB resection

Supplementary figures and tables

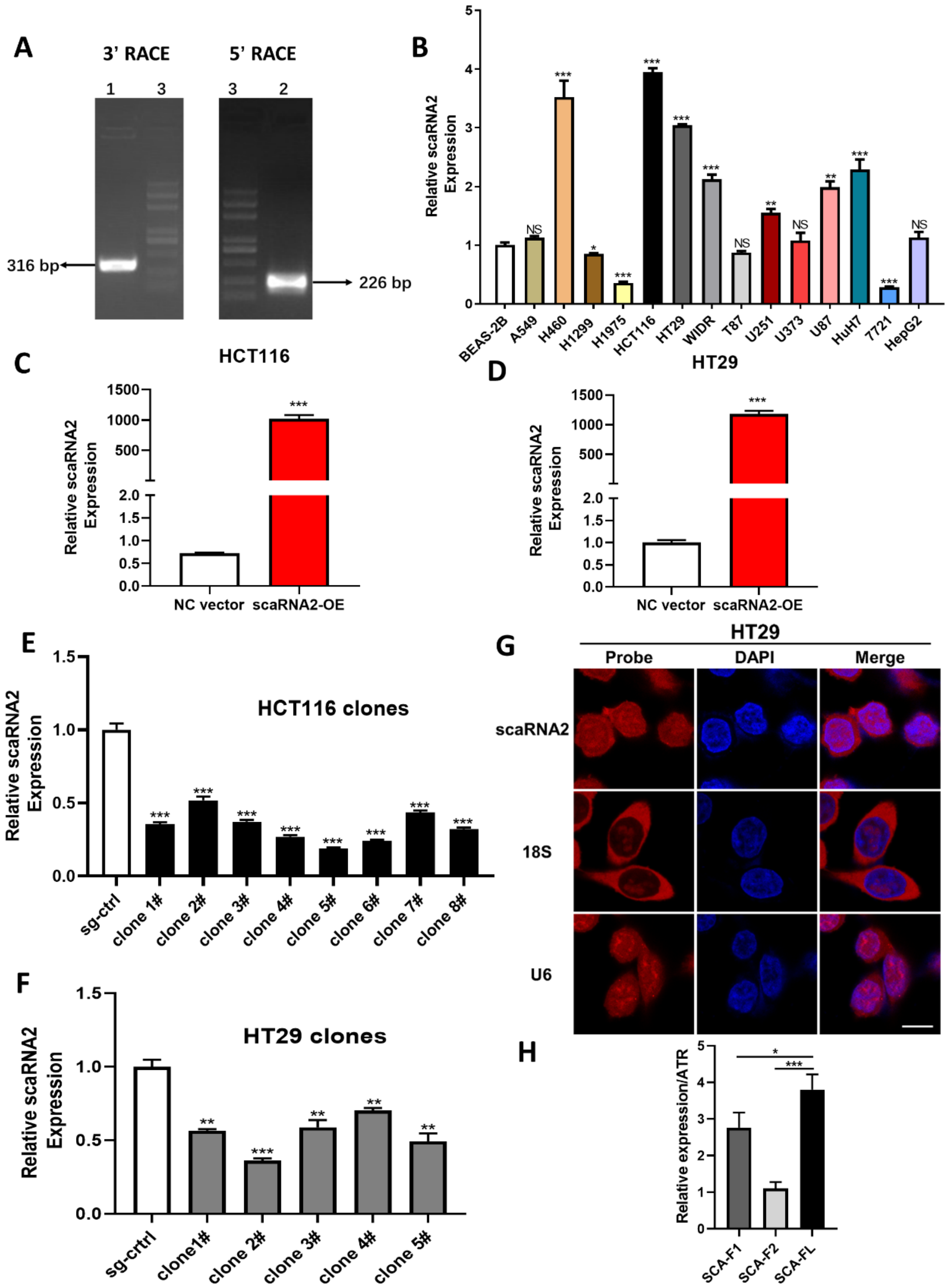

Fig. S1 Characterization of scaRNA2 and construction of stably overexpression and knockdown cell lines.

A. RACE PCR results. 1. 3' RACE result; 2. 5' RACE result; 3. Marker DL5000 Marker (M): 100, 250, 500, 750, 1000, 1500, 2000, 3000, 5000 bp; TCONS_00000860 gene splicing size: 449 bp

B. The expression of scaRNA2 across different cancer cell lines. *P < 0.05, **P < 0.01, ***P < 0.001 compared with the expression in BEAS-2B cells.

C-D. ScaRNA2 stably overexpressing HCT116 and HT29 cells were confirmed by qRT-PCR assay. ***P < 0.001 compared with the NC group

E-F. HCT116 and HT29 cell clones stably knockdown of scaRNA2 were detected by qRT-PCR. **P < 0.01, ***P < 0.001 compared with the sg-ctrl group.

G. Representative images of RNA FISH staining with a specific probe for scaRNA2 in HT29 cells. U6 small nuclear RNA and 18S ribosomal RNA were used as indicators of nuclear and cytoplasmic RNA, respectively. Scale bar: 20 μm.

H. Quantitative analysis of ATR protein in RNA pulldown experiments with scaRNA2 full-length (SCA-FL), scaRNA2 fragment 1 (SCA-F1), scaRNA2 fragment 2 (SCA-F2). *P < 0.05, ***P < 0.001 compared with the SCA-FL group.

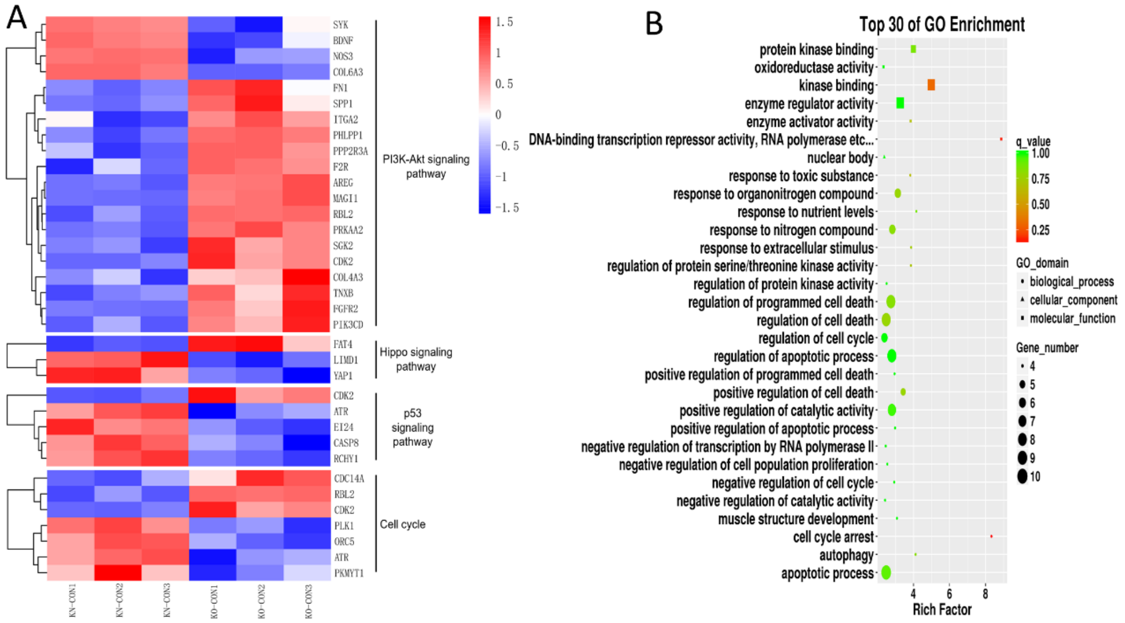

Fig. S2 Bioinformatics analysis of RNA sequencing in normal and scaRNA2 knockdown cells

A. Heat map of upregulated genes and downregulated genes in sg-ctrl and scaRNA2 knockdown cells.

B. GO analysis of the biological processes affected by scaRNA2.

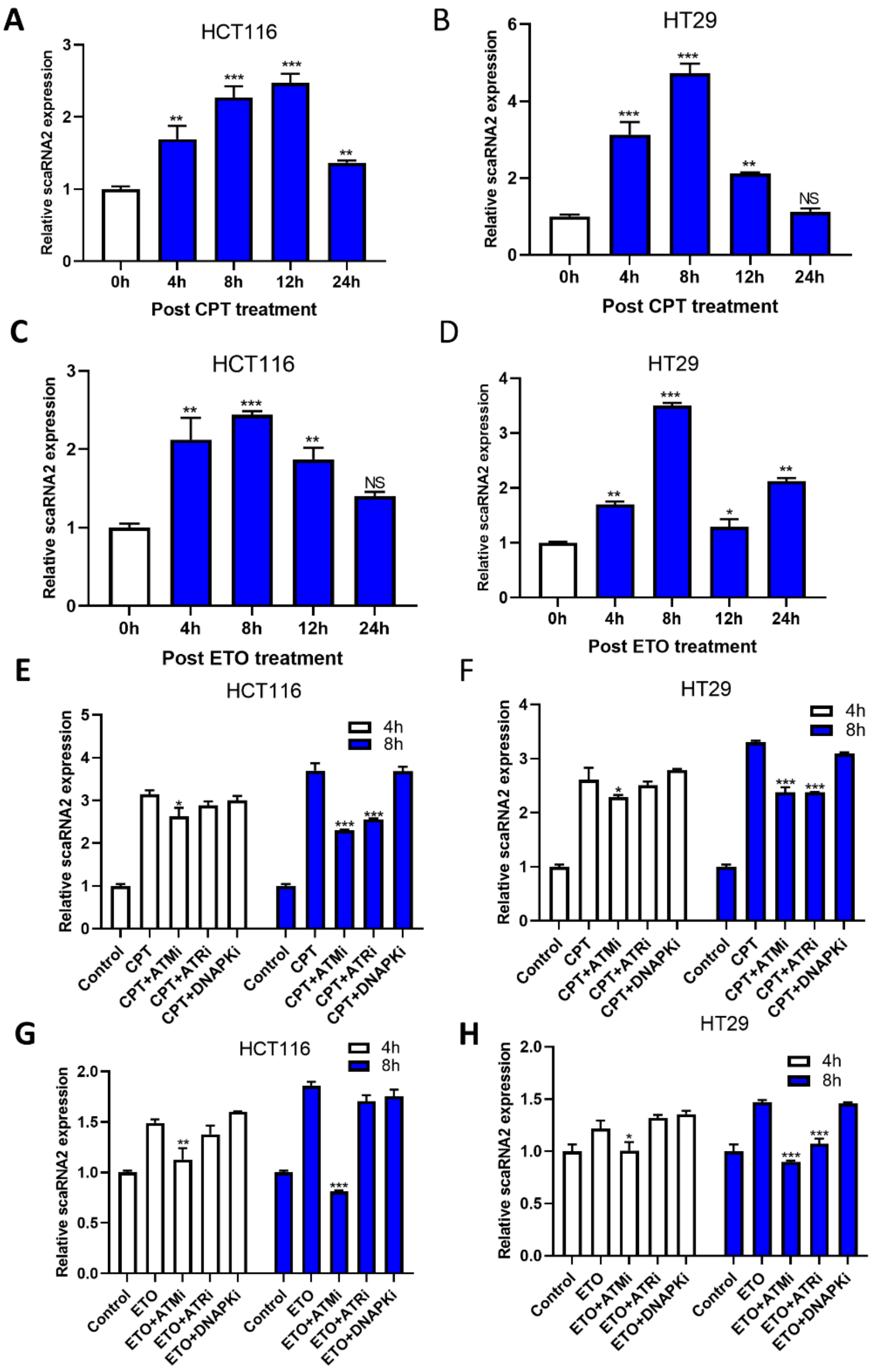

Fig. S3 ScaRNA2 is responsive to DNA damage in colorectal cancer cells.

A-B. Relative expression of scaRNA2 in HCT116 and HT29 cells detected with an RT-PCR assay at 0, 4, 8, 12 and 24 h after CPT treatment.

C-D. Relative expression of scaRNA2 in HCT116 and HT29 cells detected with an RT-PCR assay at 0, 4, 8, 12 and 24 h after ETO treatment.

E-F. ScaRNA2 expression in HCT116 and HT29 cells treated with an ATM inhibitor (KU55933, 10 μM), ATR inhibitor (VE821, 10 μM) and DNA-PKcs inhibitor (NU7441, 10 μM) at 2 h before CPT treatment.

G-H. ScaRNA2 expression in HCT116 and HT29 cells treated with an ATM inhibitor (KU55933, 10 μM), ATR inhibitor (VE821, 10 μM) and DNA-PKcs inhibitor (NU7441, 10

μM) at 2 h before ETO treatment.

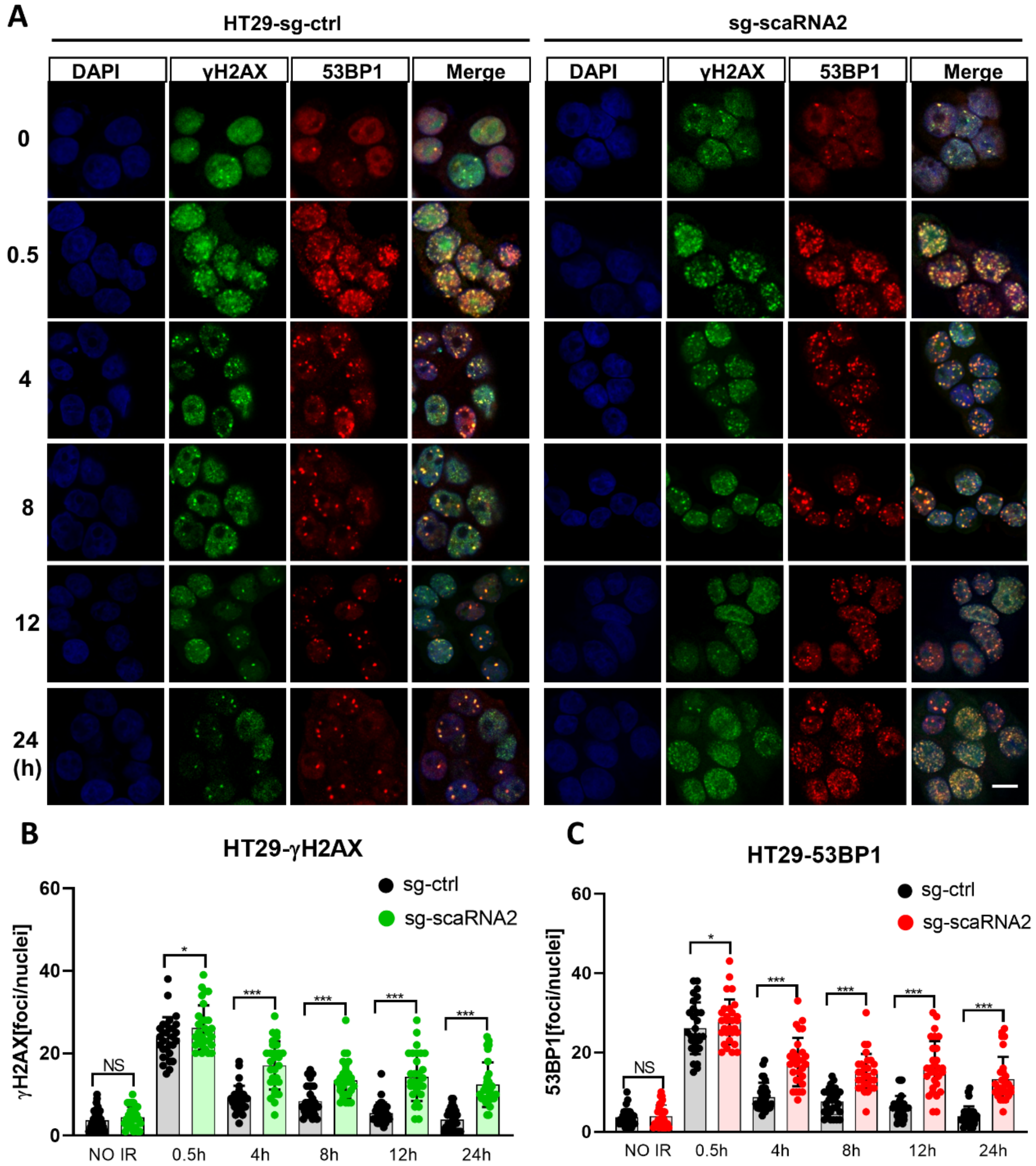

Fig. S4 ScaRNA2 knockdown impairs DNA damage repair kinetics in HT29 cells.

A. Immunofluorescence staining of γH2AX and 53BP1 in irradiated sg-ctrl and scaRNA2 knockdown HT29 cells at 0, 0.5, 4, 8, 12 and 24 h after irradiation (6 Gy). Scale bar: 20 μm.

B-C. Quantification of γH2AX and 53BP1 foci per nucleus at each time point after irradiation. ***P < 0.001, *P < 0.05 compared with the sg-ctrl group. NS, nonsignificant.

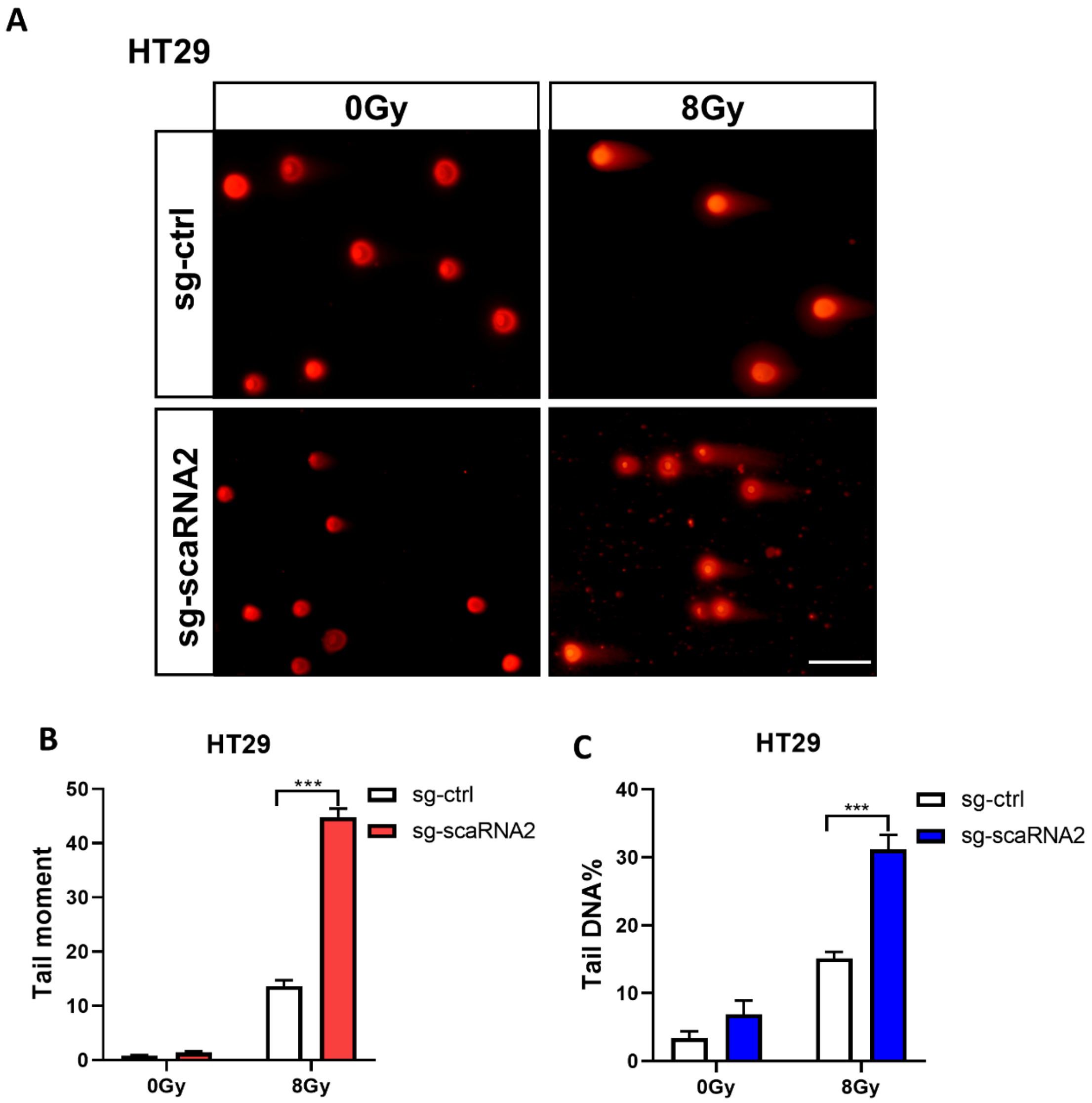

Fig. S5 ScaRNA2 knockdown resulted in more unrepair DNA damages in HT29 cells.

A. Representative fluorescence images of neutral comet assay in irradiated sg-ctrl and scaRNA2 knockdown HT29 cells (8 Gy). Scale bar: 50 μm.

B-C. Quantification of comet tails (tail moment and tail DNA) at 0h and 8 h in irradiated sg-ctrl and scaRNA2 knockdown cells (8 Gy). ***P < 0.001 compared with the sg-ctrl group.

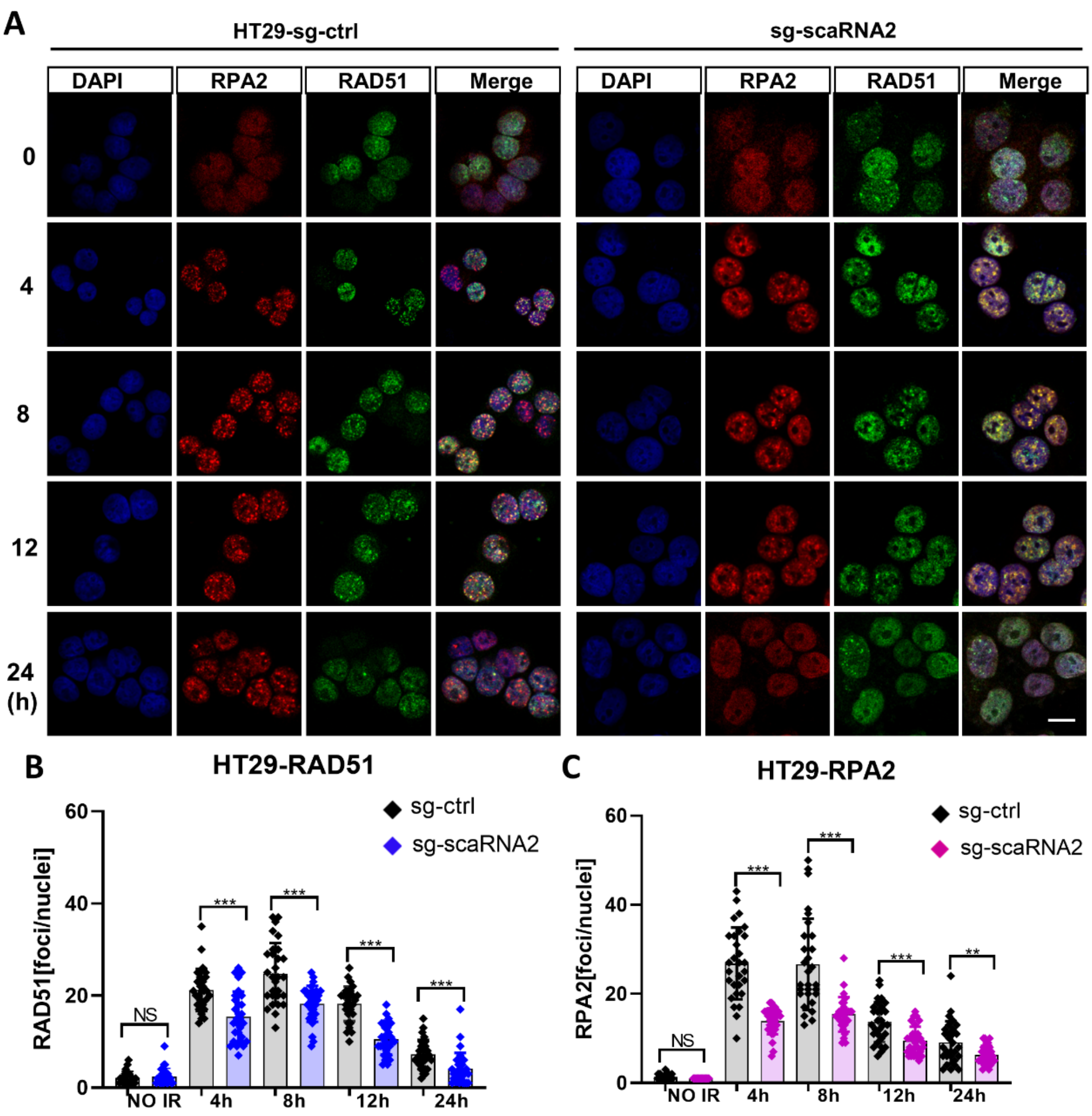

Fig. S6 ScaRNA2 knockdown inhibited the recruitment of RPA2 and RAD51.

A. Immunofluorescence staining of RPA2 and RAD51 foci in irradiated sg-ctrl and scaRNA2 knockdown HT29 cells at the indicated time points after irradiation (6 Gy). Scale bar: 20 μm.

B-C. The numbers of RAD51 foci and RPA2 foci per nucleus in irradiated HT29 cells with/without scaRNA2 knockdown. **P < 0.01, ***P < 0.001 compared with the sg-ctrl group.

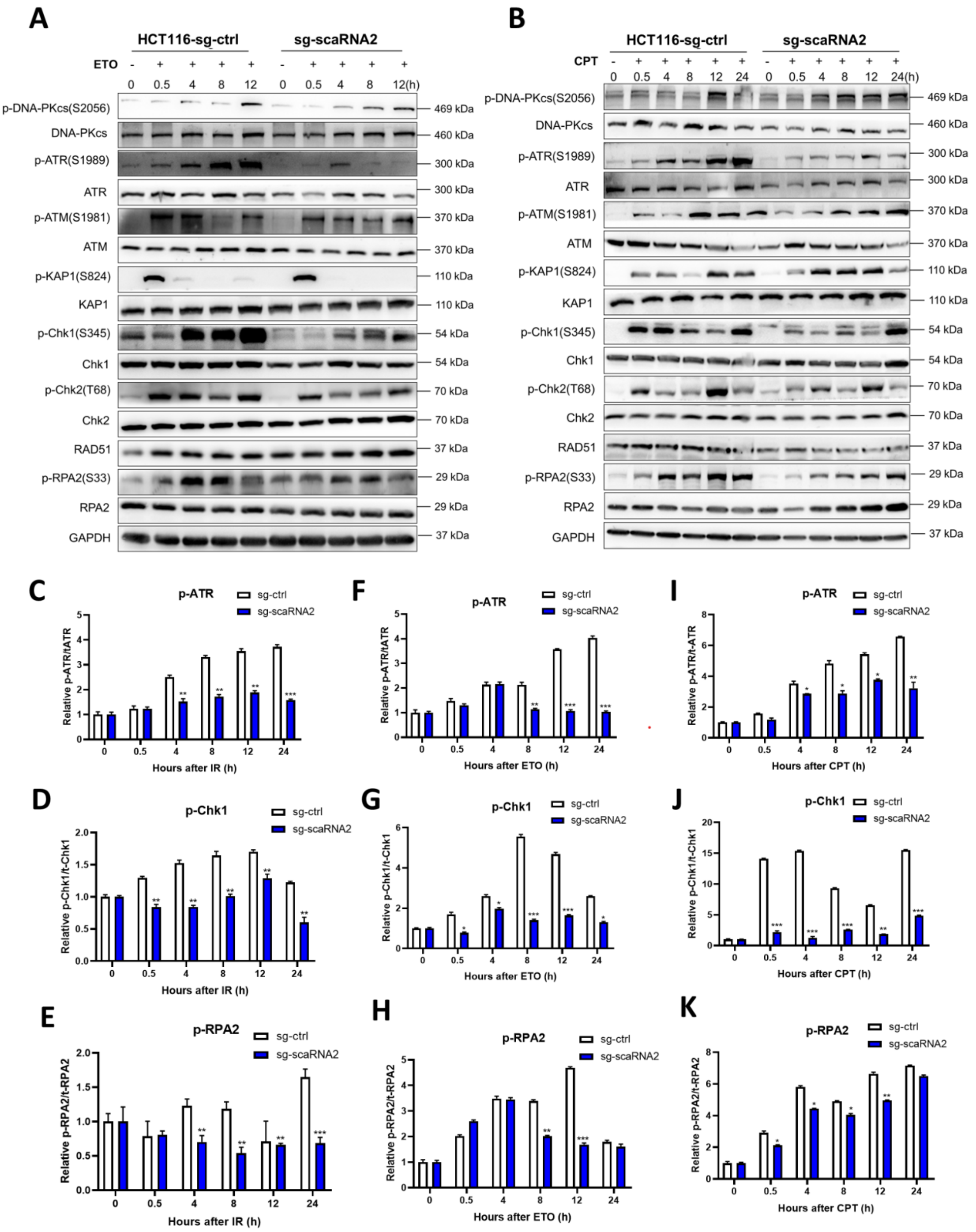

Fig. S7 ScaRNA2 is necessary for the DNA damage responses after CPT and ETO treatment.

A-B. HCT116 cells with/without scaRNA2 knockdown were treated with 100 μg/mL ETO for 4 h or 1 μM CPT for 1 h, and proteins involved in the DNA damage response were detected with western blotting assays at the indicated time points. The specific sites for phosphorylated proteins are indicated. GAPDH was used as a negative control.

C-K. Quantification of the relative density based on gray value to determine protein expression or phosphorylation. GAPDH was used as the internal control, and normalized gray value of phosphorylated protein = the grey value of phosphorylated protein/gray value of total protein. $^{*}P < 0.05$, $^{**}P < 0.01$, $^{***}P < 0.001$

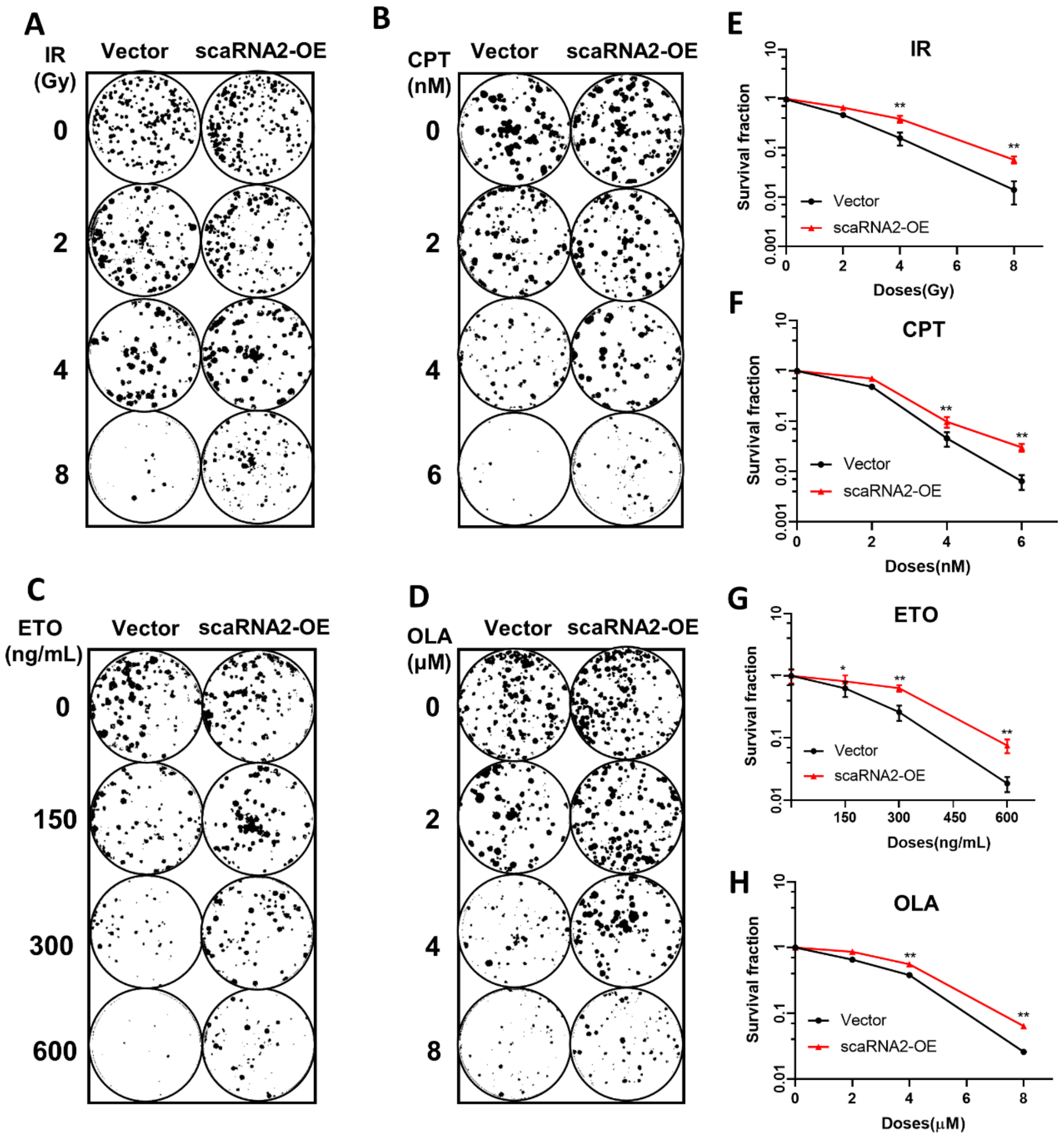

Fig. S8 Overexpression of scaRNA2 significantly increased cellular resistance to DNA damage treatments.

A-D. Clone formation in control and scaRNA2 overexpressing HCT116 cells after irradiation (A), CPT (B), Etoposide (C) or Olaparib (D) treatment at the indicated doses (concentrations).

E-H. Representative graphs are shown. The graphs depict the count number from three independent experiments. $^{*}P < 0.05$, $^{**}P < 0.01$ compared with the Vector group at the same dose of treatment.

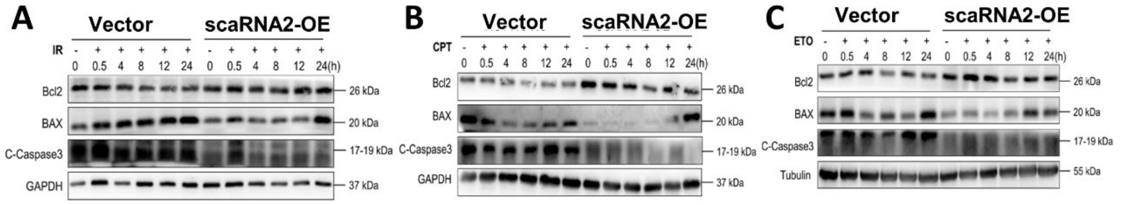

Fig. S9 Overexpression of scaRNA2 inhibits apoptosis activation by DNA damage.

A-L. Protein levels involved in the cell apoptosis signaling pathway in Vector and scaRNA2 overexpressing HCT116 cells at the indicated times after 8 Gy irradiation (A), 1 μM CPT for 1 h (B) or 100 μg/mL ETO for 4 h (C).

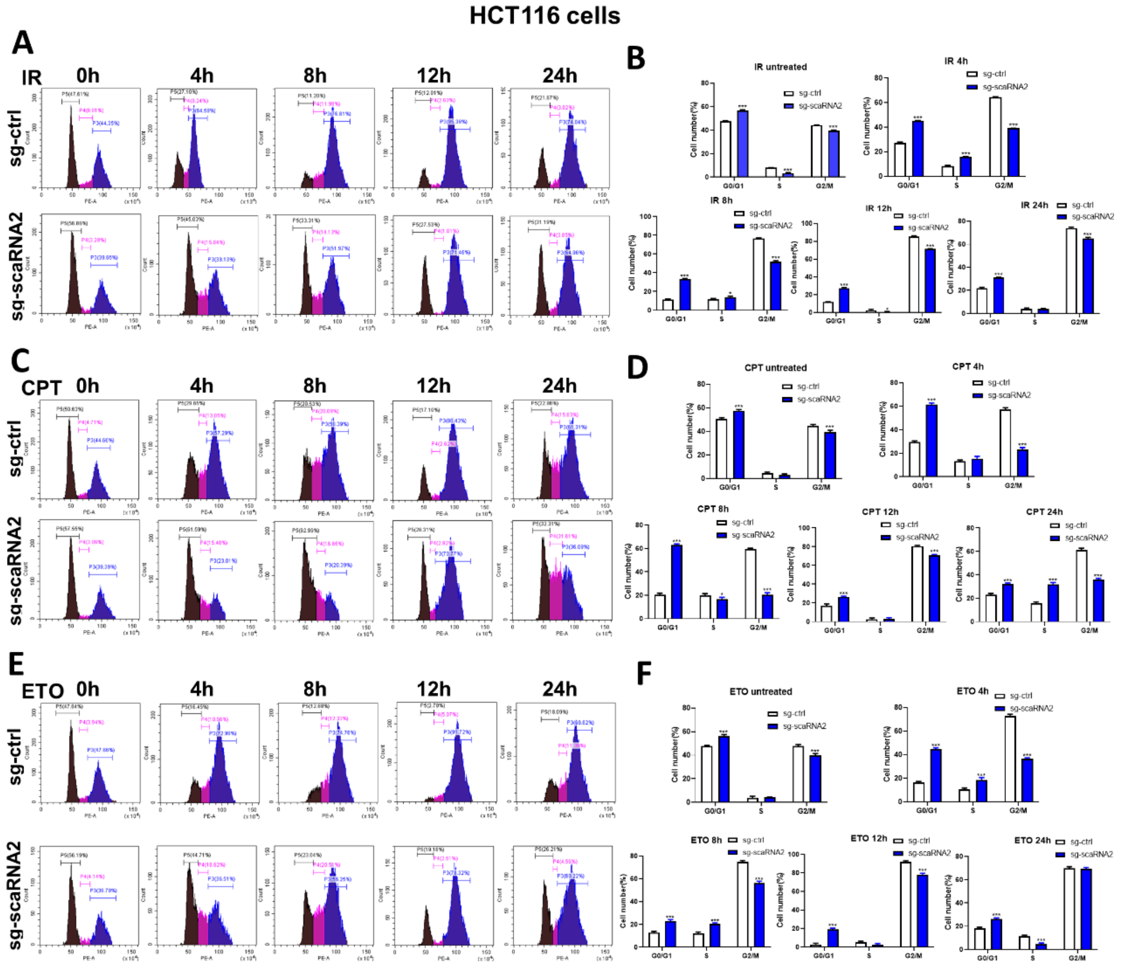

Fig. S10 ScaRNA2 knockdown inhibited DNA damage checkpoint activation in HCT116 cells.

A-F. Analysis of the cell cycle phase distribution in the G0/G1, S or G2/M phase by flow cytometry in sg-ctrl and scaRNA2 knockdown HCT116 cells at the indicated times after 8 Gy irradiation (A-B), 1 μM CPT for 1 h (C-D) or 100 μg/mL ETO for 4 h (E-F). *P < 0.05, **P < 0.01, ***P<0.001 compared with the sg-ctrl group at the same dose of treatment.

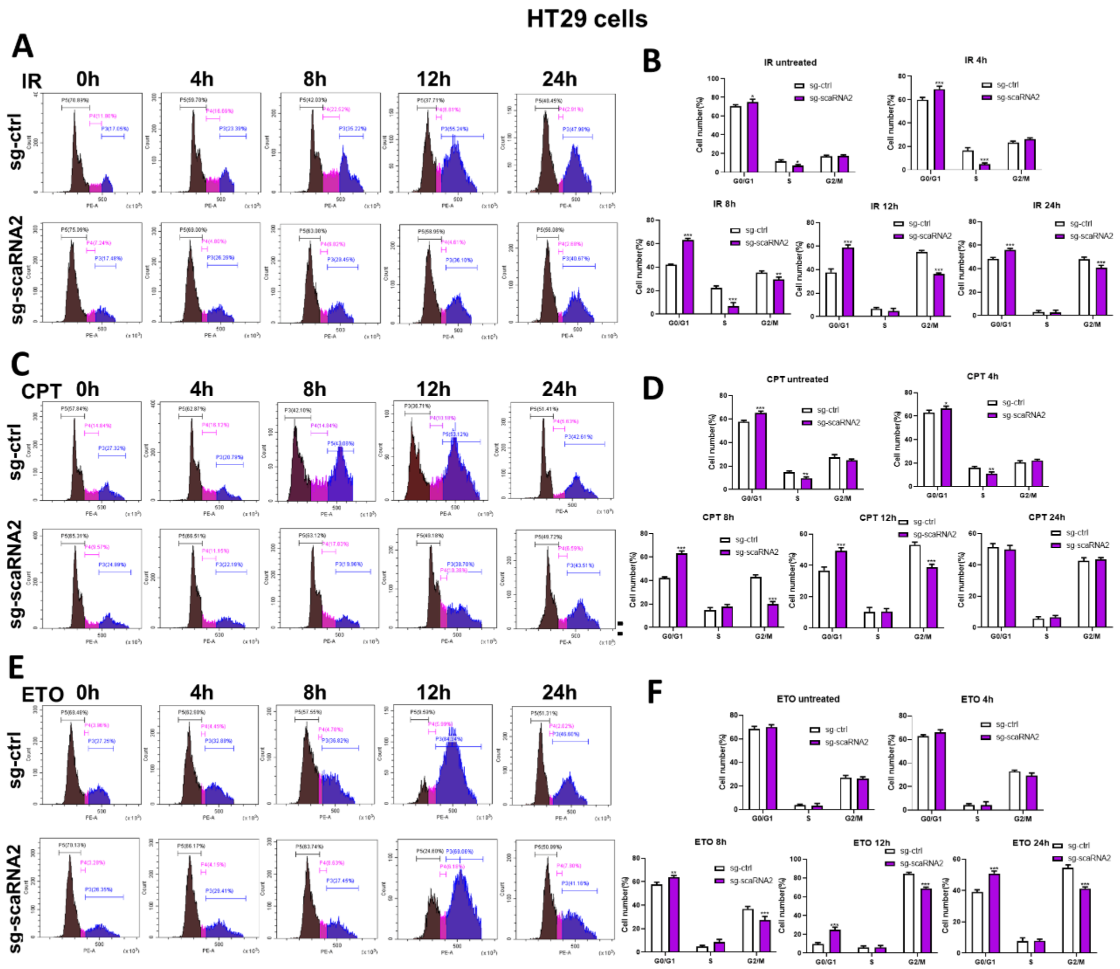

Fig. S11 ScaRNA2 knockdown inhibited DNA damage checkpoint activation in HT29 cells.

A-F. Analysis of the cell cycle phase distribution in the G0/G1, S or G2/M phase by flow cytometry in sg-ctrl and scaRNA2 knockdown HT29 cells at the indicated times after 8 Gy irradiation (A-B), 1 μM CPT for 1 h (C-D) or 100 μg/mL ETO for 4 h (E-F). *P < 0.05, **P < 0.01, ***P<0.001 compared with the sg-ctrl group at the same dose of treatment.

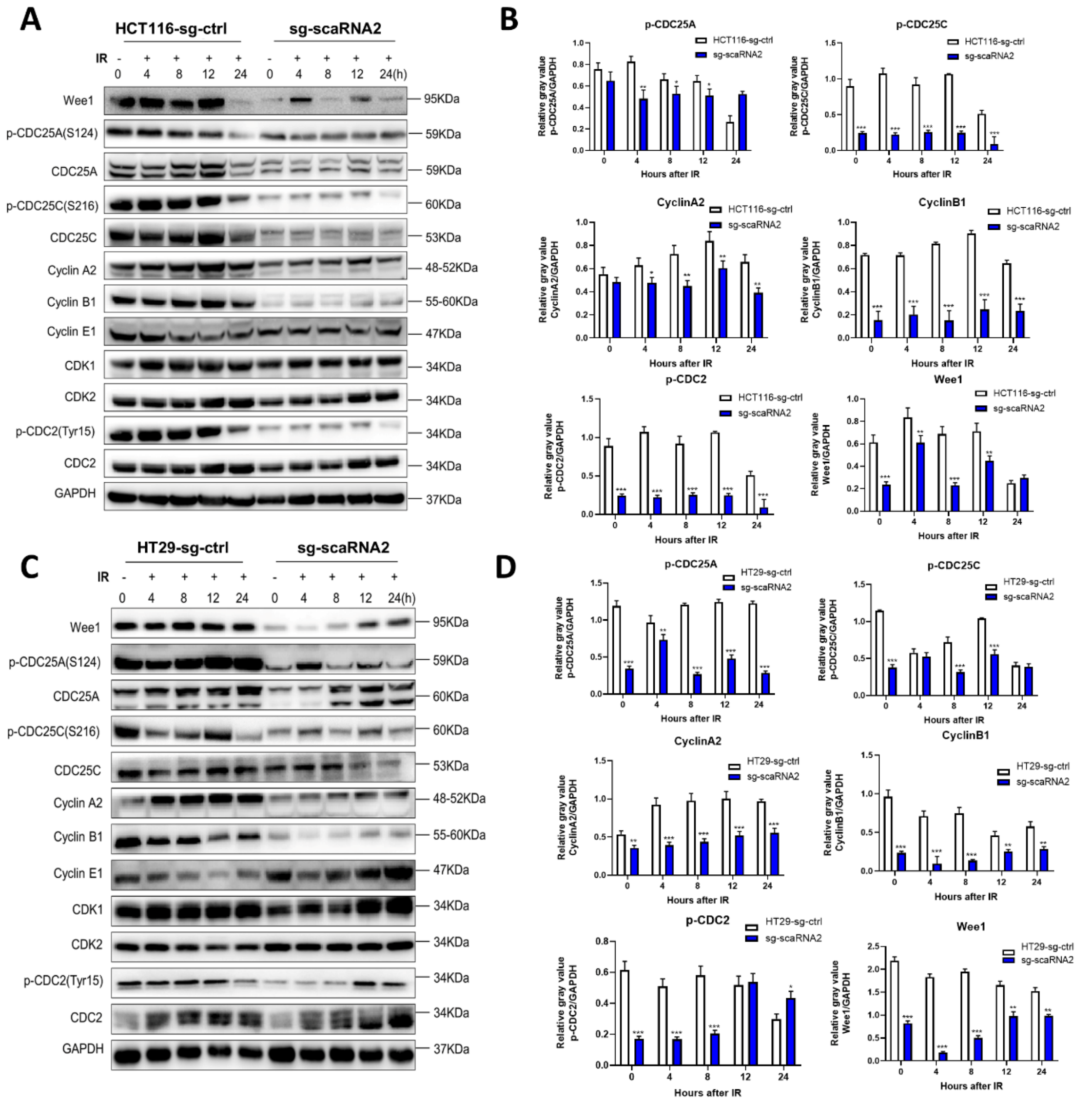

Fig. S12 Knockdown of scaRNA2 inhibits the progression of cell cycle into G2/M after irradiation.

A-D. Protein levels and quantitative analysis involved in the cell cycle signaling pathway in sg-ctrl and scaRNA2 knockdown HCT116 (A-B) or HT29 (C-D) cells at the indicated times after 8 Gy irradiation. *P<0.05, **P<0.01, ***P < 0.001 compared with the sg-ctrl group at the same dose of treatment.

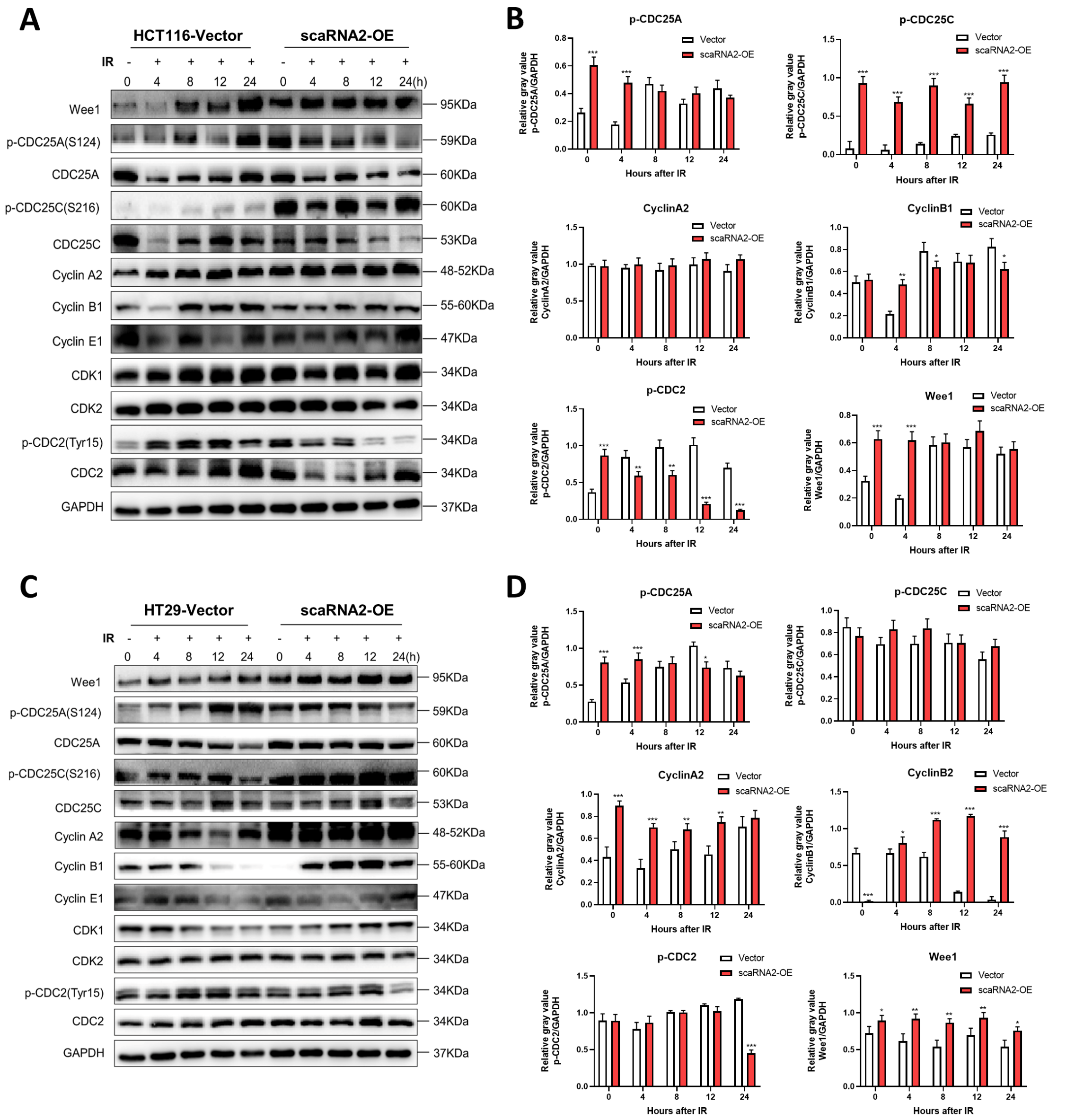

Fig. S13 Overexpression of scaRNA2 promoted cell cycle progression after irradiation.

A-D. Protein levels and quantitative analysis involved in the cell cycle signaling pathway in Vector and scaRNA2 overexpressing HCT116 (A-B) or HT29 (C-D) cells at the indicated times after 8 Gy irradiation. *P<0.05, **P<0.01, ***P < 0.001 compared with the NC group at the same dose of treatment.

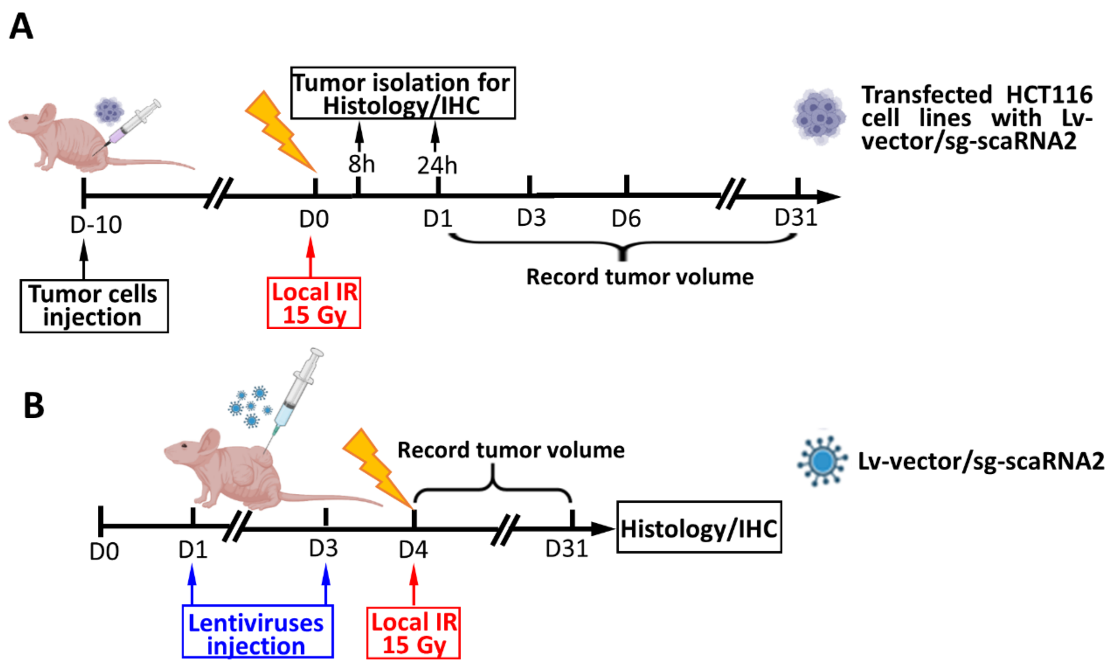

Fig. S14 Schematic illustration of local irradiation and lentivirus transfection in cell-derived xenografts (CDX, A) and patient-derived xenografts (PDX, B).

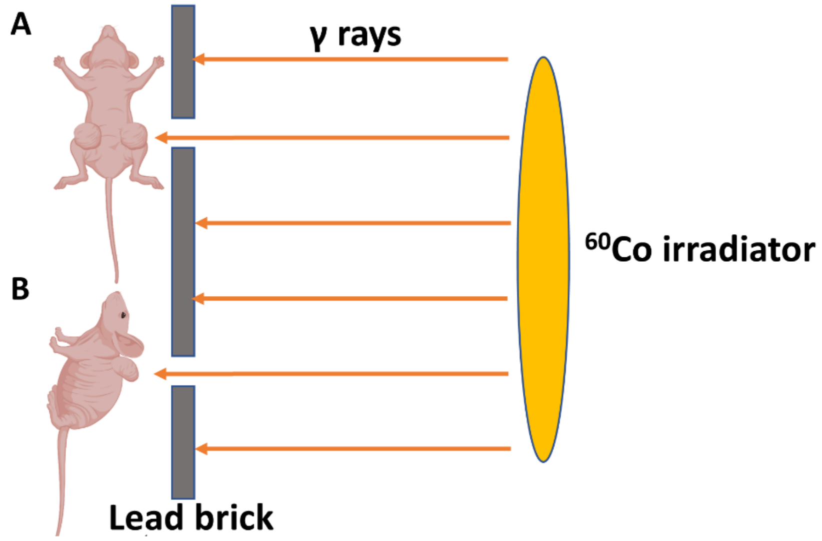

Fig. S15 Schematic diagram of local irradiation field and shielding of cell-derived xenografts (CDX, A) and patient-derived xenografts (PDX, B).

A

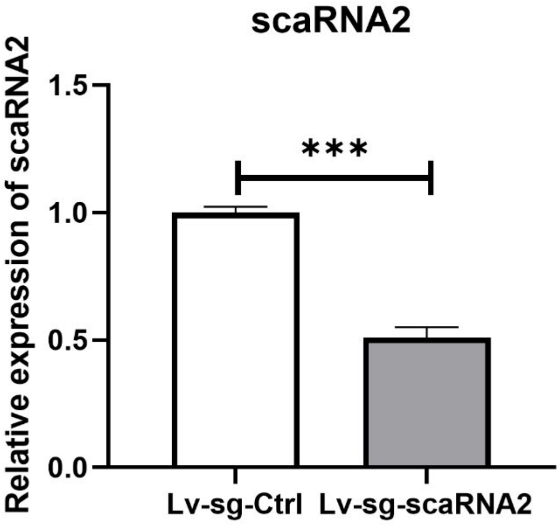

B

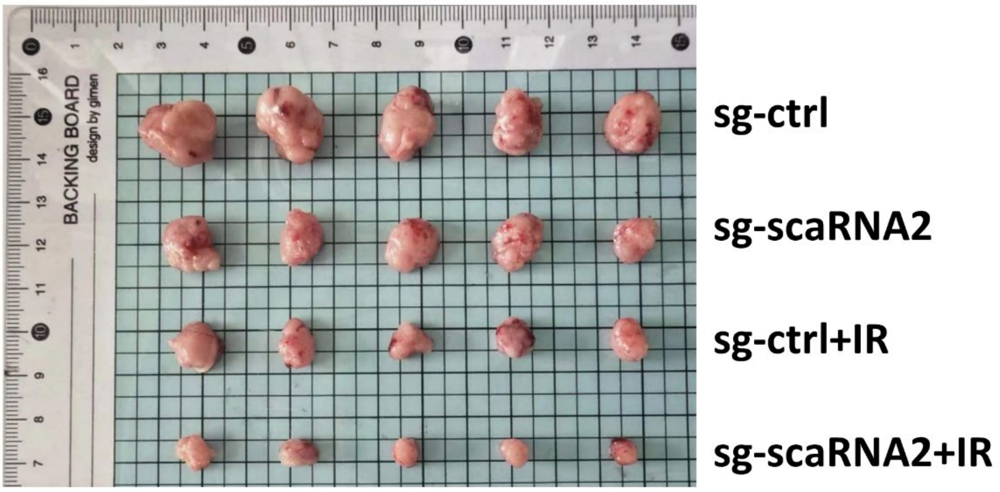

Fig. S16 Knockdown of scaRNA2 sensitized colorectal cancer to radiotherapy.

A. The knockdown efficacy of was confirmed with RT-PCR after intratumoral injection with scaRNA2 knockdown lentivirus or sg-ctrl lentivirus. ***P < 0.001 compared with the sg-ctrl group.

B. Tumor images of xenografts after 15 Gy local irradiation in four groups at the end of the experiment (n = 5).

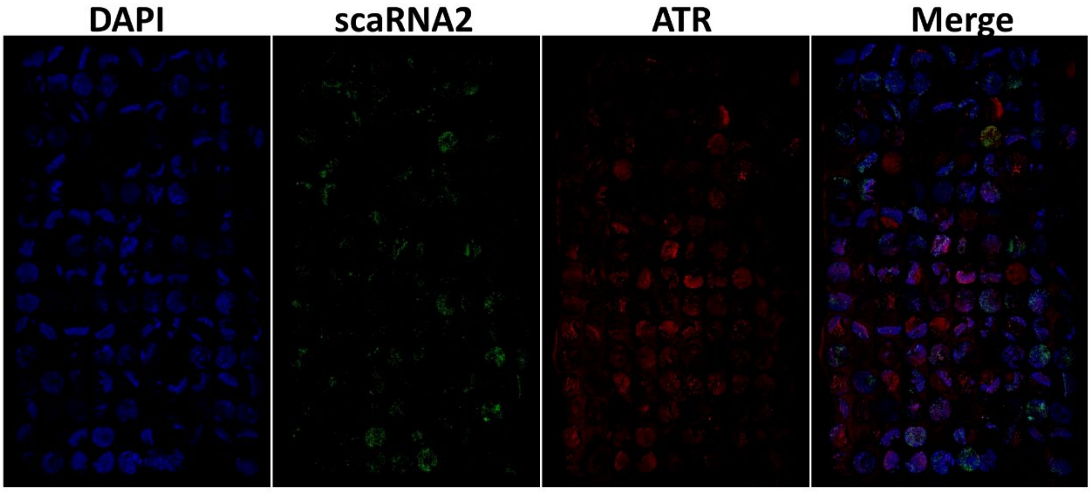

Fig. S17 Scanned image of tissues microarray including CRC patients included in our study. RNA FISH and immunofluorescence staining were performed to detect the expression of scaRNA2 and ATR, respectively.

Supplementary tables

Table S1 The cells and culture conditions.

Cell Line	Culture Conditions
**HCT116**	McCoy's 5A
**HT29**	DMEM
**WIDR**	DMEM
**T87**	DMEM
**A549**	RPMI-1640
**H460**	RPMI-1640
**H1299**	RPMI-1640
**H1975**	RPMI-1640
**BEAS-2B**	DMEM
**U251**	DMEM
**U373**	DMEM
**U87**	MEM
**HuH7**	DMEM
**7721**	DMEM
**HepG2**	MEM (minimum Eagle's medium)
**HEK 293T**	DMEM
**Hela**	DMEM
**Hela-pDDR**	DMEM plus 400 μg/ml neomycin

Table S2 List of PCR primers used in the study.

Gene ID	Primer name	Sequence (5'-3')
**NR_003023**	**scaRNA2-F**	TGGAGCGTGTTAGGCGAGT
	**scaRNA2-R**	CAAATAAGATCAAAGTGTAAGCGGG
**NR_110259**	**LINC02613-F**	CCTCAACTCTGTCCTCTGTCACTG
	**LINC02613-R**	TGGTTCTTCAAACTTCTACTCACG
**NR_109836**	**PANDAR-F**	CCCAACAAACAAGGGGTGGT
	**PANDAR-R**	GTGGCCAAAGGATCTGACGA
**NR_027471**	**LINC02893-F**	CTGGGTGGTGTCATAGTCAGGA
	**LINC02893-R**	CGGAGGTTAATGCTTTCGG
**NR_147696**	**LOC100287837-F**	GGCAACCTCGGACTCACAA
	**LOC100287837-R**	AGGAGAAGAGCCGCCAGACT
**NR_033828**	**LOC100129931-F**	ACTTCTAGCCTGGTTTCTTCCAA
	**LOC100129931-R**	TTCACTGTCTGCACTGCCTTC
**XR_001753933**	**LOC107985304-F**	AGTAATGACCTTCTGCCTTAGTGAC
	**LOC107985304-R**	CCCATTTCTGGATGTGTGTAGACT
**NR_026876**	**RNF217-AS1-F**	GCACGAACACTATTGGGAACTATG
	**RNF217-AS1-R**	GAAACTGTTTCACCTCAGATCATCA
**NR_146626**	**LINC00537-F**	TCCTCGGGTGTAGAGGGAGA
	**LINC00537-R**	GAGTTCCAGGTCCAGCGAGA
**NR_147835**	**PGLS-DT-F**	TTGGGTGATTGTCTTTGGCA

	**PGLS-DT-R**	CACTGGCTTCCCGAACTCAC
**XR_001741553**	**LINC02600-F**	GCTGATTTGTCTTGTCAGGATGT
	**LINC02600-R**	GTGCCAAACCCATCAATGCT
**XR_001746463**	**LOC107986997-F**	CACCAGACACCAGGAGTAAGGA
	**LOC107986997-R**	CAGGAATAGAAGGGAGGTCATTC
**XR_001748264**	**LOC107984338-F**	CCCGAATGTCTGGAGAGGAC
	**LOC107984338-R**	CCAGAGCTTCCTCATCACCCT
**XR_002957933**	**LOC112268179-F**	GTCAAAGTTCTGGGTGAGCTGT
	**LOC112268179-R**	CATCTTACCTTACAGATGAGGGAAC
**NR_033339**	**CTBP1-DT-F**	ACAGGCAACTGAGAACGGAGA
	**CTBP1-DT-R**	TGTGCAGACGAACACTGTGTGT
**NR_135083**	**LOC105369632-F**	AGCCTGGAGTTCCACCTACC
	**LOC105369632-R**	TGGTCAGAGGTCACTGGCAAC
**XR_932169**	**LOC105370790-F**	CTGATGGAGTCAACCTGAGGCT
	**LOC105370790-R**	TGGGCAGATGACAACATAGCTT
**NR_026880**	**MGC12916-F**	CTCCTGGGATGGTGATTCCA
	**MGC12916-R**	GCCCAAGTGTGTCTGATCTCAG
**NR_036504**	**ZNF566-AS1-F**	TGAGCCTTCTGGGAGATGTAGT
	**ZNF566-AS1-R**	GAGCAATTAAGATGACTTTCGGAG
	**GAPDH-F**	GGTCATCCATGACAACTTTGG
	**GAPDH-R**	TTCAGCTCAGGGATGACCTTG
	**U6-F**	AGAGAGATTACATGGCCCCT
	**U6-R**	CTAATGTCACGCACGATTCT
	**neomycin-F**	CGTTGGCTACCCGTGATATT
	**neomycin-R**	GCCCAGTCATAGCCGAATAG

Table S3 List of Sequences of sgRNAs and primers employed in this study

scaRNA2	sgRNAs for CRISPR	
**scaRNA2 sgRNAs**	sg1-F	CACCGCATCAGGCCCACACC
	sg1-R	TGCGGGCTGCCGGTGGCGTA
	sg2-F	CACCGGCGGAGAGCTTAAAAGTCGC
	sg2-R	AAACGCGACTTTTAAGCTCTCCGCC
	sg3-F	CACCGCGTAGGGGCGAGCACGTGAA
	sg3-R	AAACTTCACGTGCTCGCCCCTACGC

**scaRNA2 OE sequence**		GGGGGAGGCAATAGCAGAGCTCGTTTAGTGAACCGTCAGA TCGAATTCGTTTTAGGGAGGGAGAGCGGCCTGGGTCCTGG GTGTTGTGTGCGGAGCTGTGGCGTCGCGTGTGAGGCGCGT GCAGGGTGAGTGTGAGTGGACGCGTGAGTGTGTGAGTGTG CGCGCTTGGAGCGTGTTAGGCGAGTGCGTGCGCCCACCCC TGCGCCCCTCCTCCCGCTTACACTTTGATCTTATTTGATCGG ATCGTGACCCCAGCCCCGCCGGGCCGACCCGAAATGAAAA GCTCTCCTCCTGCGAAGCCCCCTCGGGGCGCTGTGCAGCG AGGCCCCTTAGGCGGCGGCCACGCTGTGGTCCCCGAGGTC CCGGAGCTGGCCCTGCGGGGCCCGGCGCTCAGAAGTGATG AATTGATCAGATAGACGAGGCCGGGCTTGTCCCCGGCCAC TGATTATCGAGGCGATTCTGATCTGGGGATCCCGCCTCCCC GCCTGGAATTAATTCTGCAGGGCGCGCCCTCGAGCTTTAAG ACCAATGACTTACAAGGCAGCTGTAGATCTTAGCCAC
**scaRNA2 (1-250bp)**	F primer	TCGTCCAACCAAACCGACTC
	R primer	TCGCTACGTGACTGGGTCATG
**scaRNA2 (251-420bp)**	F primer	TCGTCCAACCAAACCGACTC
	R primer	TCGCTACGTGACTGGGTCATG
		**RNA FISH probes (SABER-ISH)**
**scaRNA2**		5'-CGCACGCACTCGCCTAACACGCTCCAA(TTT CATCATCAT ACATCATCAT)30 -3'
	Fluorescent label probe	5'-DIG-TTATGATGATGT ATGATGATGT-3'

Table S4 List of antibodies used in the study

Antibodies	Source	Catalogue No	Species	Dilution IF	Dilution WB	Dilution IHC
**GAPDH**	Proteintech	60004-1-Ig	Mouse		1:10000	
**β-Tubulin**	Proteintech	10068-1-AP	Rabbit		1:1000	
**IgG**	Cell Signaling	2729s	Rabbit			
**p-DNA-PKcs (S2056)**	Abcam	ab124918	Rabbit		1:5000	
**DNA-PKcs**	Abcam	ab32566	Rabbit		1:1000	
**ATR**	Abcam	ab289363	Rabbit	1:500		1:50
**ATR**	Cell Signaling	13934	Rabbit			
**p-ATR (Phospho T1989)**	Abcam	ab223258	Rabbit		1:1000	
**ATM**	Abcam	ab32420	Rabbit		1:1000	

**p-ATM (Phospho S1981)**	Abcam	ab81292	Rabbit		1:50000	
**KAP1**	Abcam	ab109289	Rabbit		1:1000	
**p-KAP1 (S824)**	Abcam	ab70369	Rabbit		1:1000	
**Chk1**	Abcam	ab32531	Rabbit		1:1000	
**p-Chk1 (S345)**	Cell Signaling	2348s	Rabbit		1:1000	1:50
**Chk2**	Abcam	ab207446	Rabbit		1:1000	
**p-Chk2 (T68)**	Abcam	ab32148	Rabbit		1:1000	
**RAD51**	Abcam	ab133534	Rabbit	1:250	1:10000	1:200
**RPA2**	Abcam	ab76420	Rabbit	1:50	1:1000	1:100
**p-RPA2 (Phospho S33)**	Abcam	ab211877	Rabbit		1:1000	
**Phospho-H2AX (Ser139)**	Abcam	ab81299	Rabbit	1:250	1:5000	1:200
**Phospho-H2AX (Ser139)**	Cell Signaling	80312	Mouse	1:200		
**53BP1**	Abcam	ab175933	Rabbit	1:100		
**Mre11**	Santa Cruz	sc-135992	mouse	1:200	1:1000	
**Mre11**	Cell Signaling	4895s	Rabbit			1:100
**NBS1**	Cell Signaling	3002s	Rabbit		1:1000	
**H3**	Abcam	ab1791	Rabbit		1:1000	
**RAD50**	Abcam	ab124682	Rabbit		1:5000	
**Exo1**	Abcam	ab95068	Rabbit		1:1000	
**Exo1**	Abcam	ab106303	Rabbit	1:200		
**DNA2**	Abcam	ab220883	Rabbit	1:200		
**BLM**	Abcam	ab226945	Rabbit	1:100		
**CtIP**	Abcam	ab155988	Rabbit	1:100		
**BRAC1**	Abcam	ab213929	Rabbit	1:500		
**Bcl2**	Proteintech	12789-1-AP	Rabbit		1:1000	
**BAX**	Proteintech	50599-2-Ig	Rabbit		1:1000	
**p21**	Abcam	ab188224	Rabbit		1:1000	
**p53**	Abcam	ab26	Mouse		1:1000	
**p-p53(S15)**	Abcam	ab223868	Rabbit		1:5000	
**Cytochrome C**	Abcam	ab133504	Rabbit		1:5000	
**C-Caspase3**	Abcam	ab32042	Rabbit		1:500	
**CDC25A**	Proteintech	55031-1-AP	Rabbit		1:1000	
**CSC25A(S124)**	Abcam	ab156574	Rabbit		1:1000	
**CDC25C**	Proteintech	16485-1-AP	Rabbit		1:2000	

**CDC25C (Ser216)**	Cell Signaling	4901T	Rabbit		1:1000	
**CDK1**	Proteintech	19532-1-AP	Rabbit		1:1000	
**CDK2**	Proteintech	10122-1-AP	Rabbit		1:1000	
**Cyclin A2**	Proteintech	18202-1-AP	Rabbit		1:1000	
**Cyclin B1**	Proteintech	55004-1-AP	Rabbit		1:1000	
**CDC2**	Cell Signaling	77055T	Rabbit		1:1000	
**Wee1(Ser642)**	Cell Signaling	4910T	Rabbit		1:1000	
**Wee1**	Cell Signaling	4936T	Rabbit		1:1000	
**Cy3™ Affinipure Fab Fragment Goat Anti-Rabbit IgG (H+L)**	Jackson	111-167-003	Rabbit	1:500		
**Cy™3 AffiniPure Fab Fragment Goat Anti-Mouse IgG (H+L)**	Jackson	115-167-186	Mouse	1:500		
**Alexa Fluor 488 Affinipure Goat Anti-Mouse IgG (H+L)**	Jackson	115-545-003	Mouse	1:500		
**HRP secondary**	Servicebio	GB23301	Mouse		1:5000	
**HRP secondary**	Servicebio	GB23303	Rabbit		1:5000	

Table S5 Sequences of and primers employed in RACE experiment

		Sequence (5’-3’)
**TCONS_00000860**	m860-F	GTGCAGGGTGAGTGTGAGT
	m860-R	CGGCCTCGTCTATCTGATC
**5-RACE primer**	r860-R1	GGGGCTTCGCAGGAGGAGAGCTTTTC
	r860-R2	GGCTGGGGTCACGATCCGATCAAA
**3-RACE primer**	r860-F1	GAGTGTGTGAGTGTGCGCGCTTGGA
	r860-F2	CCCCTCCTCCCGCTTACACTTTGATC

**5-RACE obtained sequence size: 226bp**	GACTGAAGGAGTAGAAAGTTTTAGGGAGGGAGAGCGGCCTGGGTCCTGGGTGTTGTGTGCGG AGCTGTGGCGTCGCGTGTGAGGCGCGTGCAGGGTGAGTGTGAGTGGACGCGTGAGTGTGTGA GTGTGCGCGCTTGGAGCGTGTTAGGCGAGTGCGTGCGCCCACCCCTGCGCCCCTCCTCCCGC TTACACTTTGATCTTATTTGATCGGATCGTGACCCCAGCC
**3-RACE obtained sequence size: 316bp**	CCCCTCCTCCCGCTTACACTTTGATCTTATTTGATCGGATCGTGACCCCAGCCCCGCCGGGC CGACCCGAAATGAAAAGCTCTCCTCCTGCGAAGCCCCCTCGGGGCGCTGTGCAGCGAGGCCC CTTAGGCGGCGGCCACGCTGTGGTCCCCGAGGTCCCGGAGCTGGCCCTGCGGGGCCCGGCGC TCAGAAGTGATGAATTGATCAGATAGACGAGGCCGGGCTTGTCCCCGGCCACTGATTATCGA GGCGATTCTGATCTGGGCACGGCCACCGAAAAAAAAAAAAAAAAACACTGTCATGCCGTTAC GTAGCG
**TCONS_00000860 gene splice size: 449bp**	GTTTTAGGGAGGGAGAGCGGCCTGGGTCCTGGGTGTTGTGTGCGGAGCTGTGGCGTCGCGTG TGAGGCGCGTGCAGGGTGAGTGTGAGTGGACGCGTGAGTGTGTGAGTGTGCGCGCTTGGAGC GTGTTAGGCGAGTGCGTGCGCCCACCCCTGCGCCCCTCCTCCCGCTTACACTTTGATCTTAT TTGATCGGATCGTGACCCCAGCCCCGCCGGGCCGACCCGAAATGAAAAGCTCTCCTCCTGCG AAGCCCCCTCGGGGCGCTGTGCAGCGAGGCCCCTTAGGCGGCGGCCACGCTGTGGTCCCCGA GGTCCCGGAGCTGGCCCTGCGGGGCCCGGCGCTCAGAAGTGATGAATTGATCAGATAGACGA GGCCGGGCTTGTCCCCGGCCACTGATTATCGAGGCGATTCTGATCTGGGCACGGCCACCGAA AAAAAAAAAAAAAAA

Table S6 Cell densities and drug concentrations in Colony Formation Assay.

IR(Gy)	Camptothecin Dosage(nM)	Olaparib Dosage(μM)	Etoposide Dosage(ng/ml)	cell numbers
**0**	0	0	0	300
**2**	2	2	150	600
**4**	4	4	300	1200
**8**	6	8	600	2400

Table S7 Quantification of scaRNA2 positive cells in rectal cancer tissues/adjacent tissues

Number	ScaRNA2 positive cell rate in rectal cancer tissues	ScaRNA2 positive cell rate in adjacent tissues	Ratio	DFS	OS
A1	0.644286199	2.407566638	0.267608875	1737	1737
A3	2.025912839	0.27820711	7.282031142	1129	1129
A5	8.25165261	2.089442815	3.949211986	1207	1207
A7	3.424246965	1.869937164	1.831209642	1642	1642
A9	0.708670136	0.513663448	1.37963902	1555	1555
A11	2.866593164	-	-	1000	1558
A13	3.840330351	-	-	1360	1360

A15	1.781315141	0.03353829	53.11287907	780	1022
B1	0.217661692	-	-	1023	1023
B3	0.411240576	1.136484334	0.361853273	1095	1095
B5	0	2.546869473	0	932	932
B7	1.05180121	4.231795654	0.248547259	-	-
B9	-	0.431674086	-	460	640
B11	6.844444444	10.29389601	0.664903205	839	839
B13	0.060564111	0.226529071	0.267356904	520	707
B15	7.265220065	1.523784285	4.767879638	1184	1184
C1	1.56506512	0.940688776	1.663743801	741	741
C3	0.22158911	1.328903654	0.166745805	600	1354
C5	0	2.145428366	0	1352	1352
C7	0.361173815	2.73481705	0.132065074	300	1080
C9	3.142920159	0.507972344	6.187187543	1293	1293
C11	-	0.19379845	-	-	-
C13	1.002506266	2.963525836	0.338281602	1235	1235
C15	3.459799489	0.493015612	7.017626632	1080	1207
D1	2.980237154	11.7327858	0.254009338	30	1170
D3	1.952085182	4.637883008	0.420900048	1165	1165
D5	3.737988187	2.469135802	1.513885216	720	1150
D7	0.781476122	2.693498452	0.290134238	480	1138
D9	1.394101877	8.085325993	0.172423707	480	1140
D11	0.799827064	1.037344398	0.77103329	1142	1142
D13	-	1.19828816	-	-	-
D15	2.110817942	2.228642179	0.947131828	1130	1130
E1	4.112164814	-	-	945	945
E3	6.519318084	1.941319215	3.358189644	-	-
E5	5.385097057	0.170648464	31.55666878	896	896
E7	0.129032258	2.052274927	0.062872794	570	889
E9	9.917274939	1.100468718	9.011864469	1053	1053
E11	0.345565246	3.572938689	0.096717374	1053	1053
E13	-	3.688622754	-	856	856
E15	-	1.524906811	-	1060	1060
F1	4.920486436	6.558545714	0.750240473	570	1013
F3	-	0.749625187	-	782	782
F5	3.59187178	2.225491999	1.61396751	912	912
F7	1.914368053	3.602636824	0.531379694	875	875
F9	17.47930083	0.310237849	56.34161301	848	848
F11	2.209818886	2.378121284	0.929228842	839	839
F13	0.974562273	2.268339768	0.429636815	365	838
F15	6.082949309	0.304955527	19.94700463	810	840
G1	0.567680081	1.957831325	0.289953518	360	550
G3	-	1.269793845	-	30	868
G5	-	3.031169574	-	780	780

G7	8.002422355	0.848845867	9.427415113	771	771
G9	0.564221706	1.501597444	0.375747647	150	150
G11	5.366294845	2.022665328	2.653080948	190	190
G13	10.81081081	0	60	232	232
G15	0.932103611	0.407331976	2.288314363	342	342
H1	0.815620366	1.598866626	0.51012408	287	287
H3	1.277694713	5.484528442	0.232963458	-	-
H5	0.081187659	2.354188759	0.03448647	617	617
H7	2.725274725	0.242277408	11.24857141	564	564
H9	2.20501237	-	-	-	-
H11	1.418439716	0.690448792	2.054373521	459	459
H13	2.544649913	1.157957245	2.197533565	300	390
H15	9.123910939	0.703933747	12.96132055	356	356
I1	0.232693426	0.130106687	1.788481679	342	342
I3	13.5692202	1.079234973	12.57299897	26	26
I5	9.372327689	2.868788211	3.266998816	276	276
I7	7.991132693	1.367006487	5.845716731	300	383
I9	1.449275362	0.992366412	1.460423634	315	315
I11	0.527704485	1.015228426	0.519788918	206	206
I13	1.888012414	0.499621499	3.778885452	172	172
I15	-	1.351351351	-	0	127
J1	1.867113057	0.866824271	2.153969518	157	157
J3	0	3.649492584	0	-	-
J5	2.345844504	0.380662352	6.16253352	-	-
